# Gender-affirming hormone therapy preserves skeletal maturation in young mice via the gut microbiome

**DOI:** 10.1172/JCI175410

**Published:** 2024-03-26

**Authors:** Subhashis Pal, Xochitl Morgan, Hamid Y. Dar, Camilo Anthony Gacasan, Sanchiti Patil, Andreea Stoica, Yi-Juan Hu, M. Neale Weitzmann, Rheinallt M. Jones, Roberto Pacifici

**Affiliations:** 1 Division of Endocrinology, Metabolism and Lipids, Department of Medicine and; 2 Emory Microbiome Research Center, Emory University, Atlanta, Georgia, USA.; 3 Department of Biostatistics, Harvard T. H. Chan School of Public Health, Boston, Massachusetts, USA.; 4 Division of Pediatric Gastroenterology, Hepatology, and Nutrition, Department of Pediatrics and; 5 Department of Biostatistics and Bioinformatics, Emory University, Atlanta, Georgia, USA.; 6 Atlanta VA Healthcare System, Atlanta, Georgia, USA.; 7 Immunology and Molecular Pathogenesis Program, Emory University, Atlanta, Georgia, USA.

**Keywords:** Bone biology, Immunology, Bone development, Sex hormones, T cells

## Abstract

Gender-affirming hormone therapy (GAHT) is often prescribed to transgender (TG) adolescents to alleviate gender dysphoria, but the effect of GAHT on the growing skeleton is unclear. We found GAHT to improve trabecular bone structure via increased bone formation in young male mice and not to affect trabecular structure in female mice. GAHT modified gut microbiome composition in both male and female mice. However, fecal microbiota transfers (FMTs) revealed that GAHT-shaped gut microbiome was a communicable regulator of bone structure and turnover in male, but not in female mice. Mediation analysis identified 2 species of *Bacteroides* as significant contributors to the skeletal effects of GAHT in male mice, with *Bacteroides* supplementation phenocopying the effects of GAHT on bone. *Bacteroides* have the capacity to expand Treg populations in the gut. Accordingly, GAHT expanded intestinal Tregs and stimulated their migration to the bone marrow (BM) in male but not in female mice. Attesting to the functional relevance of Tregs, pharmacological blockade of Treg expansion prevented GAHT-induced bone anabolism. In summary, in male mice GAHT stimulated bone formation and improved trabecular structure by promoting Treg expansion via a microbiome-mediated effect, while in female mice, GAHT neither improved nor impaired trabecular structure.

## Abstract 2

Gender-affirming hormone therapy stimulated bone formation and improved trabecular structure in male mice, and neither improved nor impaired trabecular structure in female mice.

## Graphical abstract

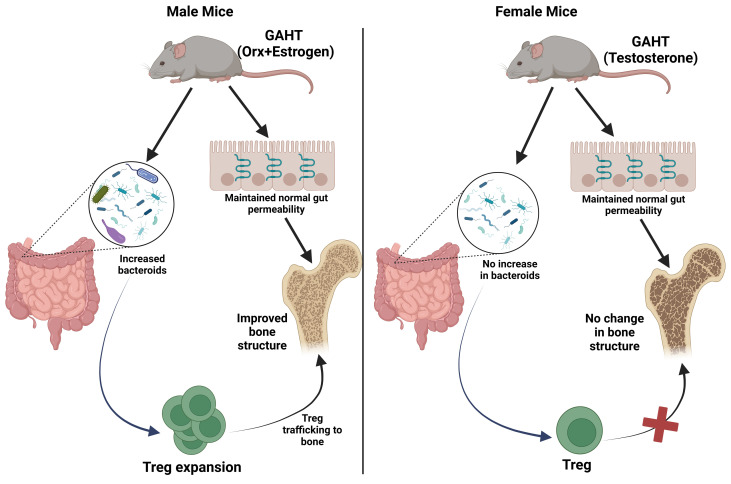

## Introduction

About 80% of people who are transgender (TG) and people who are gender nonbinary in the US are on long-term gender-affirming hormone therapy (GAHT) to alleviate gender dysphoria and/or to align their physical characteristics with their affirmed gender (1, 2). Due to the overwhelming effects of testosterone on secondary sexual characteristics, GAHT for TG women includes both drugs to block endogenous sex steroid production such as a gonadotropin-releasing hormone agonist (GnRHa), and 17-β estradiol (E2) (3, 4). By contrast, GAHT for TG men involves testosterone supplementation at doses that recapitulate testosterone concentrations in cisgender males (3, 4). TG people frequently start GAHT at 12–16 years of age, which is before the completion of skeletal development. Therefore, this practice has the potential to affect the pubertal surge in bone mass. In addition, adolescents with gender dysphoria are sometimes treated temporarily with GnRHa without cross-sex hormones to suppress puberty. In these cases, if the desire for gender identity transition persists, cross-sex hormones are added later. However, pubertal suppression without cross-sex hormone treatment may heighten bone loss or delay skeletal maturation (5).

Our understanding of the impact of GAHT on the skeleton is limited (5, 6). It was reported that pubertal suppression and GAHT did not alter bone mineral density (BMD) in TG adolescents (6), while another study showed that TG men who began sex hormones before age 14 had lower BMD compared with those who began treatment at an older age (6). Moreover, E2 supplementation was reported to prevent bone loss in TG women undergoing testosterone deprivation in one study (7) but not in another (8). Further, it was reported that testosterone was unable to maintain bone mass fully in all TG men (7), while another analysis in TG men subjected to ovariectomy followed by 2–12 years of testosterone replacement showed that testosterone preserved BMD but worsened bone geometry (9). Moreover, increased risk of osteoporosis has been reported in TG men but not in TG women on GAHT (10). Thus, the skeletal effects of GAHT in growing children and young adults remain to be comprehensively characterized.

Animal studies have also provided limited information on the effect of GAHT on skeletal development. A study on female mice concluded that testosterone supplementation maintains a stable bone mass in mice subjected to ovariectomy at 10 weeks of age, but not in mice undergoing ovariectomy at 6 weeks, an age when skeletal development is incomplete. This report concluded that estrogen is required for optimal postnatal skeletal acquisition in female mice (11). Another report revealed that in young female mice testosterone supplementation reversed the bone loss caused by puberty blockade induced by GnRHa treatment (12). In a study in female rats, testosterone supplementation partially prevented bone loss and augmented bone strength in ovariectomized animals (13). To our knowledge, the skeletal effects of GAHT in male animals have not been investigated.

The male and female gut microbiomes have known compositional differences in both humans (14, 15) and mice (16, 17), which are further modified by menopause and sex steroid therapy (18, 19). Moreover, it is now known that the gut microbiome is a pivotal regulator of postnatal skeleton maturation, bone health, and bone responsiveness to sex steroids (20–23). We reported that female germ-free mice are protected against the bone loss induced by sex steroid deprivation (21). We further reported that sex steroid deficiency increases gut permeability, allowing microbial components to activate T cells and expand TNF^+^ T cells and IL-17–producing helper CD4^+^ T cells (Th17 cells) in the intestinal mucosa, thus increasing the production of TNF and IL-17 in the intestinal lamina propria (21). An involvement of Tregs in ovariectomy-induced bone loss was reported by other laboratories, plausibly because Tregs reside in proximity with osteoclasts and prevent ovariectomy-induced bone loss (24). Moreover, Tregs are themselves regulated by estrogen (25) and testosterone (26) and have been shown to increase bone formation (27, 28) and reduce bone resorption (29). Menopause, gut permeability, inflammation, and bone loss have been linked in mice (21) and humans (30). Together, this evidence suggests that modifications of the gut microbiome induced by sex steroids may mediate, in part, the skeletal effects of GAHT. Mechanistically, compositional changes of the gut microbiome and increased gut permeability may affect the skeleton via regulation of immune cell activation in the gut mucosa and the migration of immune cells from the gut mucosa to the BM.

In the current study, we investigated the effects of GAHT on microbiome composition and bone structure in young male and female mice. We report that E2 supplementation improved bone trabecular structure in orchiectomized (orx) male mice while testosterone supplementation did not affect skeletal maturation in eugonadic female mice. We show that the beneficial effects of GAHT on the skeleton of male mice are mediated, in part, by modifications in gut microbiome composition, including a higher prevalence of *bacteroides*, a taxon which induces an expansion of intestinal and BM Tregs and bone formation.

## Results

### Effects of GAHT on trabecular bone structure, bone turnover, and gut permeability.

**Figure 1:**
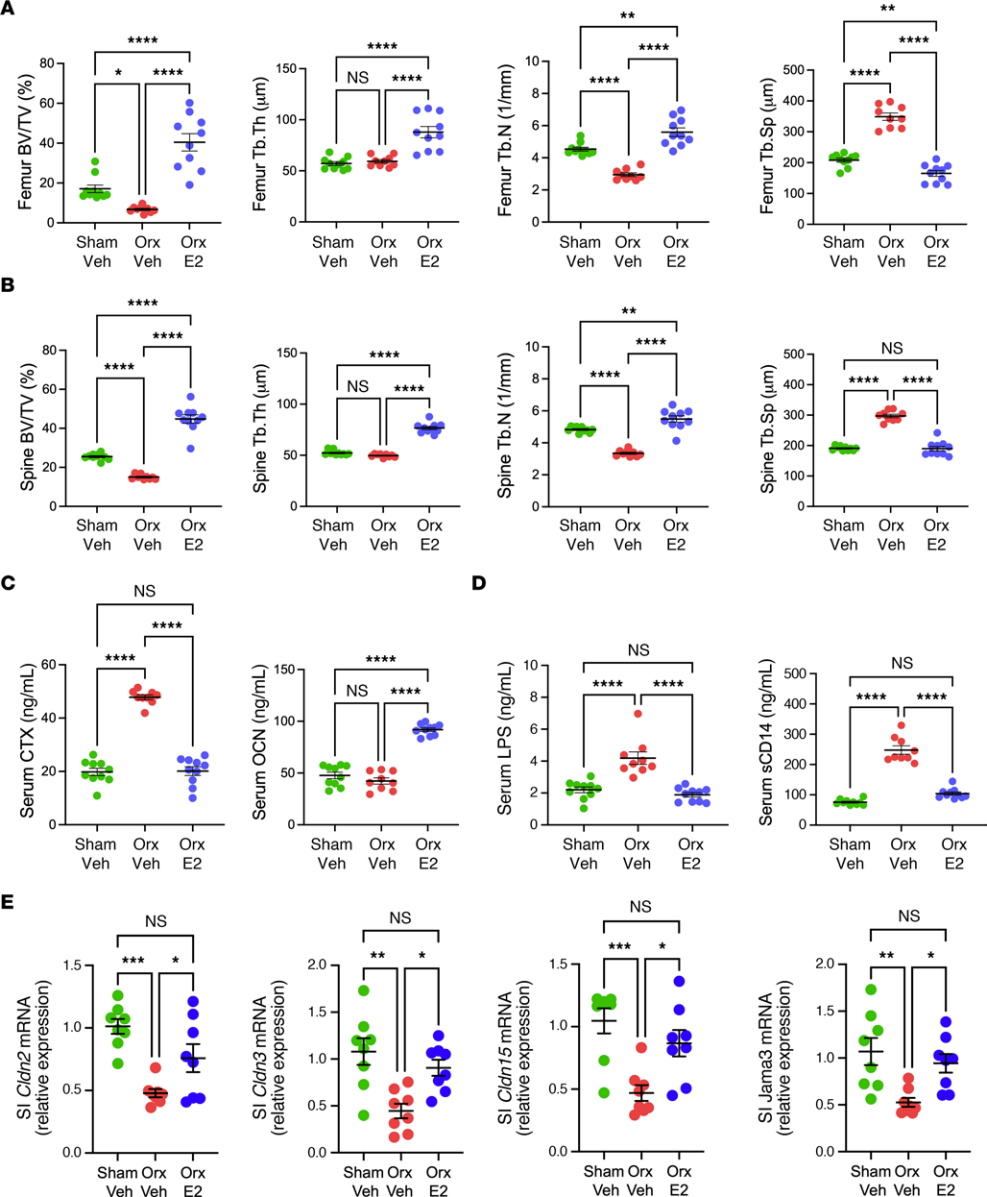
Effect of GAHT on indices of femoral and spinal trabecular structure, serum markers of bone turnover, and gut permeability in male mice. Mice received GAHT (E2, 6.4 μg/wk) or vehicle (sesame oil) for 10 weeks, from 6–16 weeks of age. Bone, serum, and gut permeability parameters were measured at endpoint. (**A**) Micro-CT indices of femoral trabecular structure. (**B**) Micro-CT indices of spinal (L4) trabecular structure. (**C**) Serum CTX and OCN levels. (**D**) Serum levels of the gut permeability indices LPS and sCD14. (**E**) SI mRNA levels of Claudin 2, 3, 15, and Jam3. All data were normally distributed and were analyzed by 1-way ANOVA and post hoc tests applying Bonferroni’s correction for multiple comparisons. *n* = 8–10 mice/group. Data were expressed as Mean + SEM. *P < 0.05, **P < 0.01, ***P < 0.001, and ****P < 0.0001. Veh, vehicle.

To model GAHT used by TG women, male mice were orx at 5 weeks of age. Orx is typically used as an alternative to treatment with GnRHa to ablate testosterone in male mice. Orx mice were then treated with weekly subcutaneous injections of either 17-β estradiol E2 benzoate dissolved in 100 μl sesame oil, at the dose used in humans allometrically adjusted to the average C57BL/6 mouse bodyweight (6.4 μg/wk) (11), or control vehicle comprising of equal volume of PBS dissolved in 100 μl sesame oil. E2 or vehicle were started 1 week after orx and continued for 10 weeks. Additional control mice were subjected to sham-orx and treated with weekly subcutaneous injections of sesame oil (vehicle) for 10 weeks starting 1 week after surgery. To model GAHT used by TG men, female mice were treated with weekly subcutaneous injections of testosterone in the allometrically weight-adjusted amount used for humans undergoing GAHT (31 μg/wk dissolved in 100 μl sesame oil) (11) once a week for 10 weeks starting at 6 weeks of age. Control female mice were treated with weekly subcutaneous injections of sesame oil (vehicle). Ovariectomy was not performed in female mice, since GAHT in TG men only involves supplementation of testosterone, a hormone that blocks endogenous estrogen production. All mice were sacrificed at 16 weeks of age, upon completion of 10 weeks of GAHT. Femurs and the fourth lumbar vertebra (L4) were harvested, and indices of trabecular structure determined by in vitro micro-CT scanning. Compared with male sham-operated controls, vehicle-treated orx mice had lower femoral and spinal bone volume/total volume fraction (BV/TV), lower trabecular number (Tb.N), higher trabecular separation (Tb.Sp) and similar trabecular thickness (Tb.Th) (Figure 1, A and B). E2 treatment not only prevented the changes in indices of trabecular volume and structure induced by orx but significantly improved volume and structure over that of sham vehicle mice (Figure 1, A and B). Mechanistic studies revealed that serum CTX, a marker of bone resorption, was higher in vehicle-treated orx mice than in sham-operated controls and E2-treated orx mice (Figure 1C). Confirming earlier reports (31, 32), serum osteocalcin (OCN), a marker of bone formation, did not increase in response to orx. However, orx-E2 mice had significantly higher OCN levels than sham vehicle or orx vehicle mice (Figure 1C). These data indicate that in male orx mice, E2 treatment not only prevented trabecular bone loss, but exerted a net bone anabolic effect driven by increased bone formation.

Estrogen deficiency is known to increase gut permeability in mice (21) and humans (30). Loss of gut barrier integrity is a key upstream mechanism by which estrogen deficiency causes bone loss (21, 30). By contrast, the effects of testosterone deprivation and testosterone cross-hormone therapy on gut permeability are unknown. Thus, blood samples were collected upon completion of 10 weeks of GAHT. In male mice, orx resulted in increased serum levels of LPS and sCD14, both established markers of gut permeability (33, 34) (Figure 1D). The leaky gut phenotype resulting from orx was not detected in E2-treated orx mice (Figure 1D). These data demonstrated that in male mice, testosterone deficiency increased gut permeability and that E2 supplementation restored a normal tight barrier function. Mechanistic studies revealed that orx and E2 treatment altered the transcript levels of the tight junction proteins Claudin 2, 3, and 15, and of JAM3 (Figure 1E), which have all been shown to modulate intestinal barrier integrity (35, 36).

**Figure 2:**
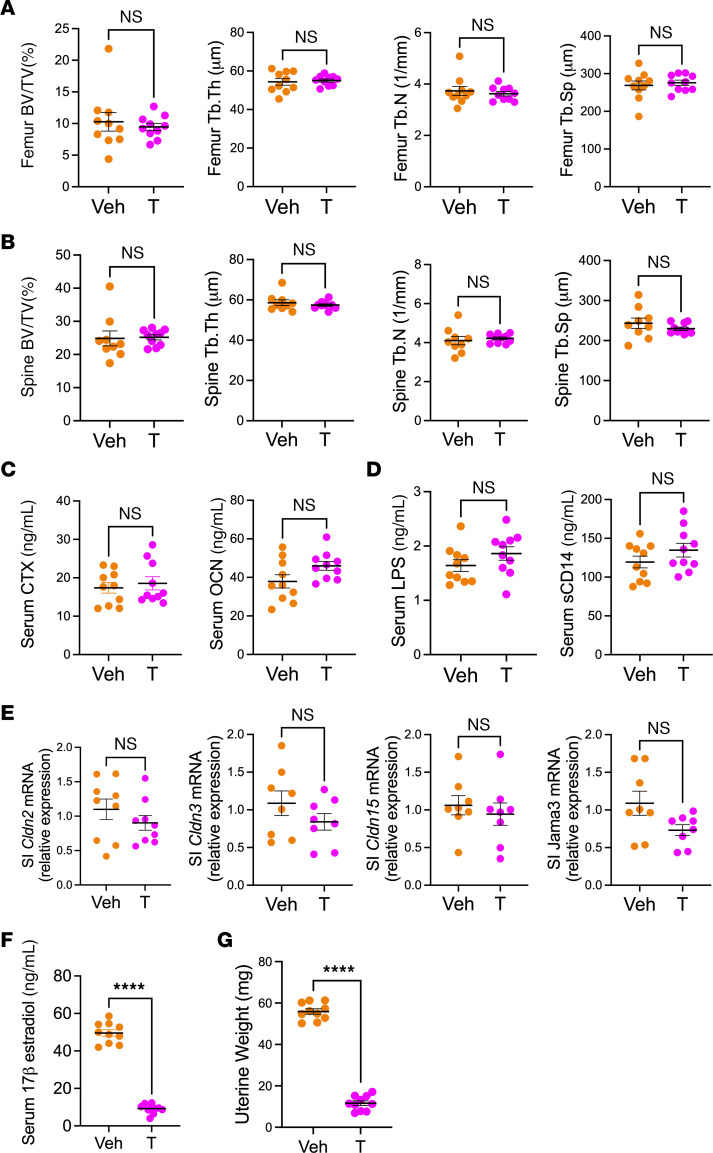
Effect of GAHT on indices of femoral and spinal trabecular structure, serum markers of bone turnover, gut permeability, serum E2 levels, and uterus weight in female mice. Mice received GAHT (Testosterone, 31 μg/wk) or vehicle (sesame oil) for 10 weeks, from 6–16 weeks of age. Bone, serum, and gut permeability parameters were measured at sacrifice. (**A**) Micro-CT indices of femoral trabecular structure. (**B**) Micro-CT indices of spinal (L4) trabecular structure. (**C**) Serum CTX and OCN levels. (**D**) Serum levels of the gut permeability indices LPS and sCD14. (**E**) SI mRNA levels of Claudin 2, 3, 15, and Jam3. (**F**) Serum E2. (**G**) Uterine weight. All data were normally distributed and were analyzed by unpaired *t* tests. *n* = 8–10 mice/group. Data were expressed as Mean + SEM. ****P < 0.0001. Veh, vehicle.

In eugonadic female mice, testosterone treatment did not alter femoral and spinal BV/TV, Tb.Th, Tb.N, and Tb.Sp (Figure 2, A and B), serum levels of CTX and OCN (Figure 2C), markers of gut permeability (Figure 2D), nor the transcript levels of the gut barrier proteins Claudin 2, 3 and 15, and JAM3 (Figure 2E). Moreover, in female mice, testosterone treatment decreased serum E2 levels and uterus weight (Figure 2, F and G). These findings demonstrated that, in female mice, testosterone treatment blunted estrogen production and was as effective as endogenous estrogen in preventing trabecular bone loss, increasing bone turnover, and preventing leaky gut phenotype.

### Compositional differences of the microbiome of male and female mice.

**Figure 3:**
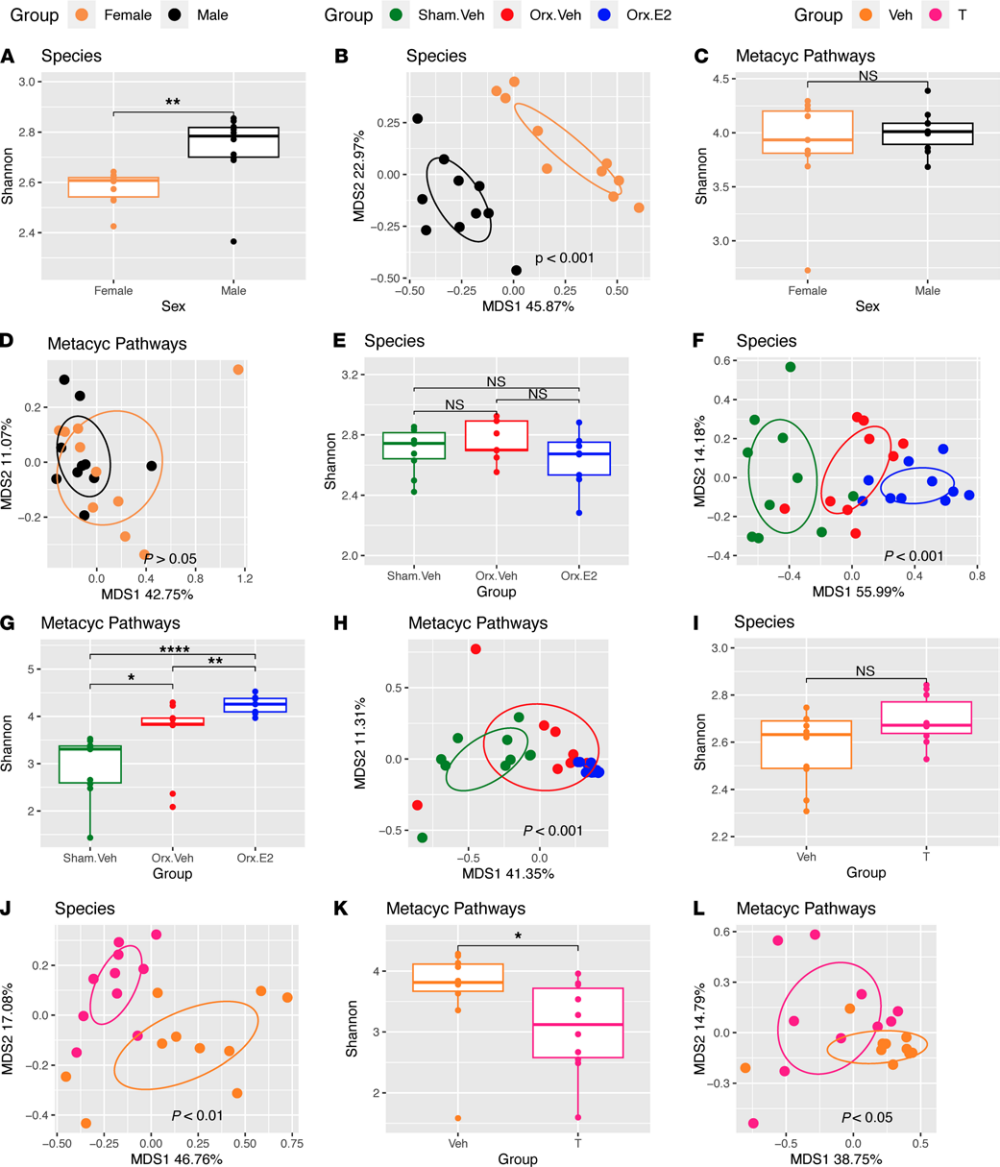
Analysis of stool microbiome before and after 4 weeks of GAHT in male and female mice. (**A**) Bacterial species α diversity analysis in untreated 6-week-old male and female mice. (**B**) Principal coordination analysis (PCoA) of bacterial species in untreated 6-week-old male and female mice. (**C**) MetaCyc pathways diversity analysis in untreated 6-week-old male and female mice. (**D**) MetaCyc pathways PCoA in untreated 6-week-old male and female mice. (**E**). Bacterial species α diversity analysis in GAHT-treated male mice. (**F**) Principal coordination analysis (PCoA) of bacterial species in GAHT-treated male mice. (**G**) MetaCyc pathways α diversity analysis in GAHT-treated male mice. (**H**) MetaCyc pathways PCoA in GAHT-treated male mice. (**I**). Bacterial species α diversity analysis in GAHT-treated female mice. (**J**) Principal coordination analysis (PCoA) of bacterial species in GAHT-treated female mice. (**K**) MetaCyc pathways α diversity analysis in GAHT-treated female mice. (**L**) MetaCyc pathways PCoA in GAHT-treated female mice. In panels **A**, **C**, **E**, **G**, **I**, and **K**, α diversity analysis was conducted by calculating the Shannon diversity index by gender. The *P* values were generated from the Wilcoxon-rank-sum test. The box shows the median and Q1–Q3 interquartile range, the bars show the minimum and maximum values. In panels **B**, **D**, **F**, **H**, **J**, and **L** PCoA plots were based on the Bray-Curtis distance metrics. The *P* values were generated from the PERMANOVA test. *n* = 9–10 mice per group. *P < 0.05, **P < 0.01, and ****P < 0.0001. Veh, vehicle.

Because sex steroids regulate postnatal skeletal maturation (37) and the composition of the gut microbiome (18), it is possible that gut microbiome compositional differences may also contribute to the development of differences in bone structure between male and female mice. To investigate the effects of sex on the composition of the gut microbiome, stool samples were collected from 6-week-old untreated male and female mice before initiation of GAHT. These samples were analyzed by metagenomic sequencing to yield data on the abundance of individual microbial species and functional features with bacterial genomes. We quantified overall community complexity (α diversity) and compositional similarity between samples (β diversity). At baseline, male and female mice had significant differences in overall community complexity as measured by Shannon diversity (Figure 3A) and microbial species (Figure 3B). A total of 37 bacterial species were differentially abundant between sexes at baseline, with the largest sex-associated species differences being in the abundance of *prevotella*, *muribaculaceae*, and *duncaniella* (Supplemental Figures 1 and 2; supplemental material available online with this article; https://doi.org/10.1172/JCI175410DS1)). These findings demonstrated the existence of a sex differences in the composition of the gut microbiome of young male and female mice. However, despite the notable differences in species composition, the functional profiles of the microbiome of male and female mice (as measured by Metacyc pathway relative abundance (38) were similar, both in overall complexity (Figure 3C) and in composition (Figure 3D). Moreover, at FDR ≤ 0.1, there were no metabolic pathways associated with sex, confirming that male and female stool microbiotas at baseline are functionally similar.

### GAHT induces compositional and functional changes in the microbiome.

Analysis of stool collected from male mice after 4 weeks of GAHT revealed significant differences in α diversity between sham vehicle and orx-E2 mice (Figure 3E). In addition, β diversity was significantly different in the 3 groups of mice (Figure 3F), reflecting significant differences in the relative abundances of 53 treatment-associated bacterial species (Supplemental Figures 3 and 4). MetaCyc pathway analysis showed that both castration and E2 treatment in male mice were associated with increased pathway complexity, as measured by Shannon diversity, and significant differences in β diversity (Figure 3, G and H). Treatment was associated with the differential activity of 49 metabolic pathways within the gut microbiome. The 19 most significant metabolic pathways associations are outlined in Supplemental Figure 5.

In female mice, β diversity was significantly different between the 2 treatment groups, while α diversity was not (Figure 3, I and J). Moreover, the relative abundance of 7 bacterial species was associated with treatment groups (Supplemental Figure 6). Functional analysis revealed that pathway complexity was decreased in testosterone-treated female mice (Figure 3K). In addition, there were overall differences in functional β diversity between the 2 groups (Figure 3L), as measured by Metacyc pathways. However, no specific metabolic pathway was significantly different between the 2 treatment groups.

Together, these data demonstrate that GAHT induced compositional and functional changes to the microbiome of both male and female mice. However, GAHT-induced changes in micro-CT indices of bone structure were observed only in male mice, suggesting that the specific modification of the gut microbiome induced by GAHT may be relevant for postnatal skeletal maturation in male, but not in female, mice. In support of this hypothesis, we found that BV/TV, the main micro-CT index of trabecular volume and structure, was associated with 8 bacterial species (Supplemental Figure 7) and 7 metabolic pathways (Supplemental Figure 8) in male mice. By contrast, no significant associations between BV/TV, bacterial species, or metabolic pathways were detected in female mice.

### GAHT-induced microbiome compositional changes contribute to the effects of GAHT on bone structure.

To directly determine if the gut microbiome contributes to the effects of GAHT on bone, fecal microbiota transfer (FMT) experiments were conducted as depicted in Supplemental Figures 9 and 10 to assess whether the skeletal phenotype of donor mice was transmissible to recipient mice via the microbiome. Firstly, fecal microbiota donor male mice were subjected to sham operation or orx at 5 weeks of age. Donor mice were supplemented with either vehicle or E2 for 4 weeks starting a week after surgery. Female donor mice were treated with vehicle or testosterone for 4 weeks starting at 6 weeks of age. Stools were collected from both male and female donor mice at the end of the treatment period and utilized for FMTs. Groups of 4-week-old FMT-recipient male and female mice were treated with broad-spectrum antibiotics (1 mg/mL ampicillin, 0.5 mg/mL vancomycin, 1 mg/mL neomycin sulfate, 1 mg/mL metronidazole dissolved in water) for 1 week to deplete the microbiome (39). Thereafter, recipient male mice were subjected to sham operation or orchiectomy. After 1 week, sham-orx mice were started on vehicle, while the 2 groups of orx mice were treated with either vehicle or E2 for 10 weeks. A week after completion of the antibiotic treatment, recipient female mice were treated with vehicle or testosterone for 10 weeks. In addition, 2 days after completion of the antibiotic treatment, a length of time sufficient for antibiotic clearing (39), male and female recipient mice were subjected to FMTs by gavaging liquid suspensions of stool samples from donor mice for 10 weeks. FMTs were repeated 3 times during the first week and once a week thereafter to assure that the microbiome of donor mice was maintained in recipient mice. This design allowed the transfer of fecal material from each group of donor mice into each group of sex-matched recipient mice. Herein, we refer to mice colonized with fecal material from a donor from a different treatment group as ‘discordant’ mice, whereas mice colonized with fecal material from a donor of the same treatment group are referred to as ‘concordant’ mice.

To investigate if FMTs successfully altered the microbiome composition of recipient mice, the stools of orx-vehicle mice with concordant or discordant microbiome were collected at the end of the experiments and the microbiome sequenced. Analysis at the species level revealed significant differences in α diversity and β diversity in the 3 groups of recipient mice (Supplemental Figure 11, A and B). MetaCyc pathway analysis showed similarities in α diversity between the 3 groups of recipient mice (Supplemental Figure 11C) but confirmed the existence of significant differences in β diversity (Supplemental Figure 11D). These findings indicated that FMTs successfully induced modifications to the compositions and function of the microbiome in orx-vehicle recipient mice.

**Figure 4:**
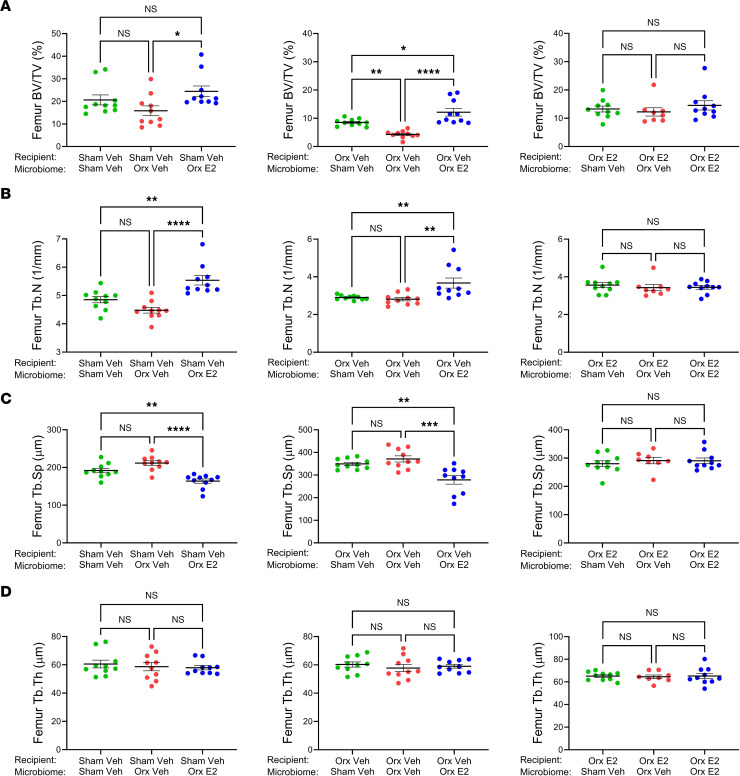
Microbiome transfer by FMT altered femoral indices of trabecular volume and structure in male recipient mice. (**A**) BV/TV, (**B**) Tb.N, (**C**) Tb.Sp, (**D**) Tb.Th were measured by micro-CT at endpoint**.** Liquid suspensions of stools collected at 4 weeks of GAHT from donor mice were gavaged into recipient mice. GAHT-treated recipient mice were sacrificed 10 weeks after treatment. *n* = 8–10 mice/group. All data were normally distributed and were analyzed by 1-way ANOVA and post hoc tests applying Bonferroni’s correction for multiple comparisons. Data were expressed as Mean + SEM. *P < 0.05 **P < 0.01, ***P < 0.001, and ****P < 0.0001. Veh, vehicle.

Micro-CT analysis of femurs harvested from recipient mice at the end of the experiment revealed that BV/TV of sham-vehicle recipients with concordant microbiome was similar to the BV/TV of sham-vehicle recipients with discordant microbiome (Figure 4A, left panel). However, sham-vehicle recipients with orx-E2 microbiome had higher BV/TV than those with orx-vehicle microbiome (Figure 4A, left panel). The BV/TV of orx-vehicle recipient mice with concordant microbiome was lower than the BV/TV of orx-vehicle recipients with discordant microbiome (Figure 4A, middle panel). Furthermore, orx-vehicle recipients with orx-E2 microbiome had higher BV/TV than those with sham-vehicle microbiome (Figure 4A, middle panel). Conversely, orx-E2 recipients with concordant or discordant microbiome had similar BV/TV values (Figure 4A, right panel). Analysis of indices of trabecular structure revealed that Tb.N and Tb.Sp of sham-orx recipient mice were altered by transfer of donor orx-E2 microbiome (Figure 4, B and C, left panels). Similarly, Tb.N and Tb.Sp of orx-vehicle recipients were altered by the transfer of microbiomes from orx-E2 donors (Figure 4, B and C, middle panels). Orx-E2 recipients with concordant or discordant microbiomes had similar Tb.N, and Tb.Sp values (Figure 4, B and C, right panels). FMTs from any group of donors did not alter Tb.Th in any group of recipients (Figure 4D). These findings demonstrate that E2 altered microbiomes from male donor mice had the capacity to modify bone volume and structure of recipient male mice. Micro-CT analysis of L4 yielded similar results, where data confirmed that transfer of microbiomes from E2-treated donors into orx-vehicle recipients altered BV/TV, Tb.N, and Tb.Sp values (Supplemental Figures 12).

**Figure 5:**
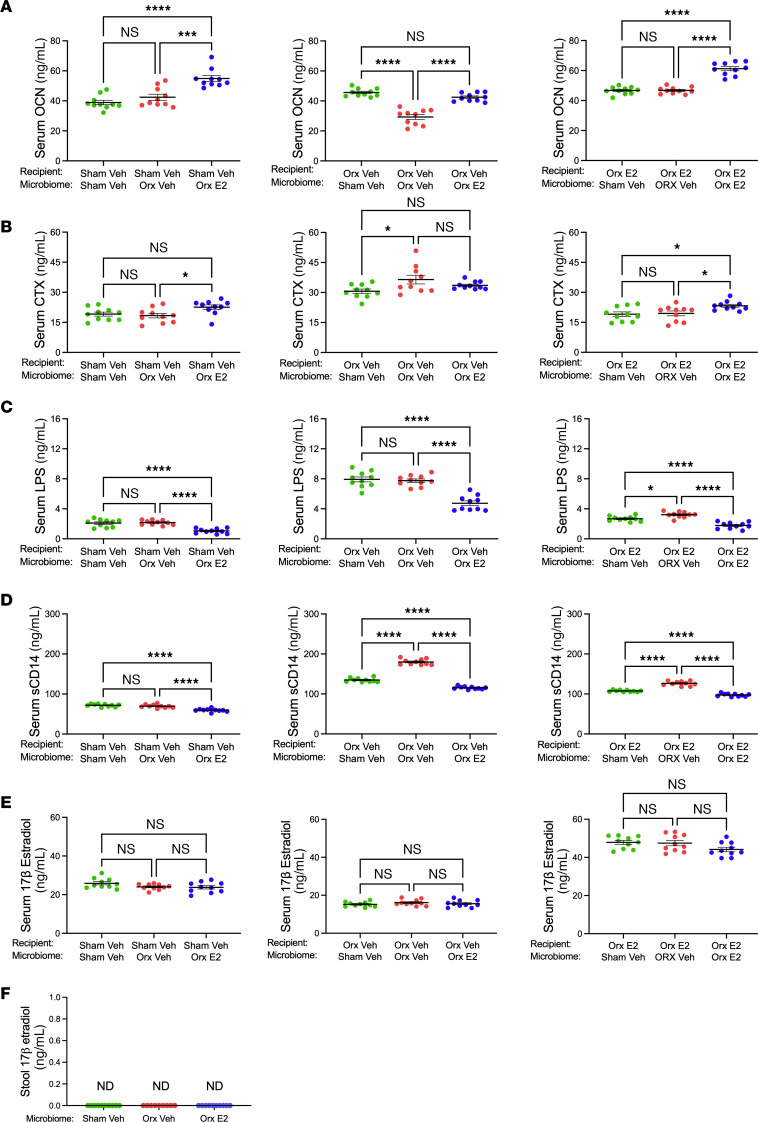
Microbiome transfer by FMT alters markers of bone turnover and gut permeability in recipient mice without affecting E2 levels. (**A**) Serum OCN, (**B**) Serum CTX, (**C**) serum LPS, (**D**) Serum sCD14, (**E**) serum E2 in recipient mice. (**F**) Stool E2 in donor mice. Factors were measured in different groups at endpoint. *n* = 10 mice/group. All data were normally distributed and were analyzed by 1-way ANOVA and post hoc tests applying Bonferroni’s correction for multiple comparisons. Data were expressed as Mean + SEM. *P < 0.05, ***P < 0.001, and ****P < 0.0001.

Differences in serum OCN levels were found between sham-orx recipients with concordant microbiome and those with orx-E2 microbiome (Figure 5A left panel). Moreover, sham-vehicle recipients with orx-E2 microbiomes had higher OCN than those with orx-vehicle microbiomes (Figure 5A, left panel). Orx-vehicle recipients with concordant microbiomes had lower levels of serum OCN compared with those with discordant microbiomes (Figure 5A middle panel). OCN levels in orx E2-treated recipients with concordant microbiomes were higher than in those with discordant microbiomes (Figure 5A, right panel). Serum CTX levels were similar in sham-orx recipients with concordant or discordant microbiomes (Figure 5B, left panel). Due to coupling of bone resorption with bone formation, sham-vehicle recipients with orx-E2 microbiomes had higher CTX levels than those with orx-vehicle microbiomes (Figure 5B, left panel). Orx-vehicle recipients with concordant microbiomes had higher levels of serum CTX than orx recipients with sham-vehicle microbiomes (Figure 5B, middle panel). Due to coupling, CTX in orx E2-treated recipients with concordant microbiomes was higher than in those with discordant microbiomes (Figure 5B, right panel).

Analysis of markers of gut permeability in sham-vehicle male recipient mice with concordant microbiomes showed similar levels of LPS and sCD14 as sham-vehicle male recipients with microbiomes from orx-vehicle mice, but higher levels of these permeability markers than those with orx-E2 microbiomes (Figure 5, C and D, left panels). Orx-vehicle recipient mice with concordant microbiomes had higher LPS levels than those with orx-E2 microbiomes (Figure 5C, middle panel), and higher sCD14 levels than those with either sham-orx or orx-E2 microbiomes (Figure 5D, middle panel). Orx-E2 recipient mice with concordant microbiomes had lower LPS and sCD14 levels than those with all groups of discordant microbiomes (Figure 5, C and D, right panels).

To investigate whether absorption of stool estrogen by recipient mice might have contributed to the effects of FMTs, E2 was measured in the serum of recipient mice and stools of donor mice. We found that transfer of microbiome from E2-treated orx donors did not increase serum levels of E2 in any group of recipient mice (Figure 5E). Moreover, E2 was found to be undetectable in the stools of donor mice (Figure 5F). Together, these findings demonstrate that the GAHT-altered microbiome of male donor mice has the capacity to modify bone volume and structure, bone turnover, and gut permeability in recipient male mice. By contrast, in female mice, microbiome transfers did not alter indices of femoral and spinal trabecular volume and structure, bone turnover, or gut permeability in recipient mice (Supplemental Figures 13 and 14).

### GAHT regulates the frequency of Tregs in Peyer’s patches and BM.

**Figure 6:**
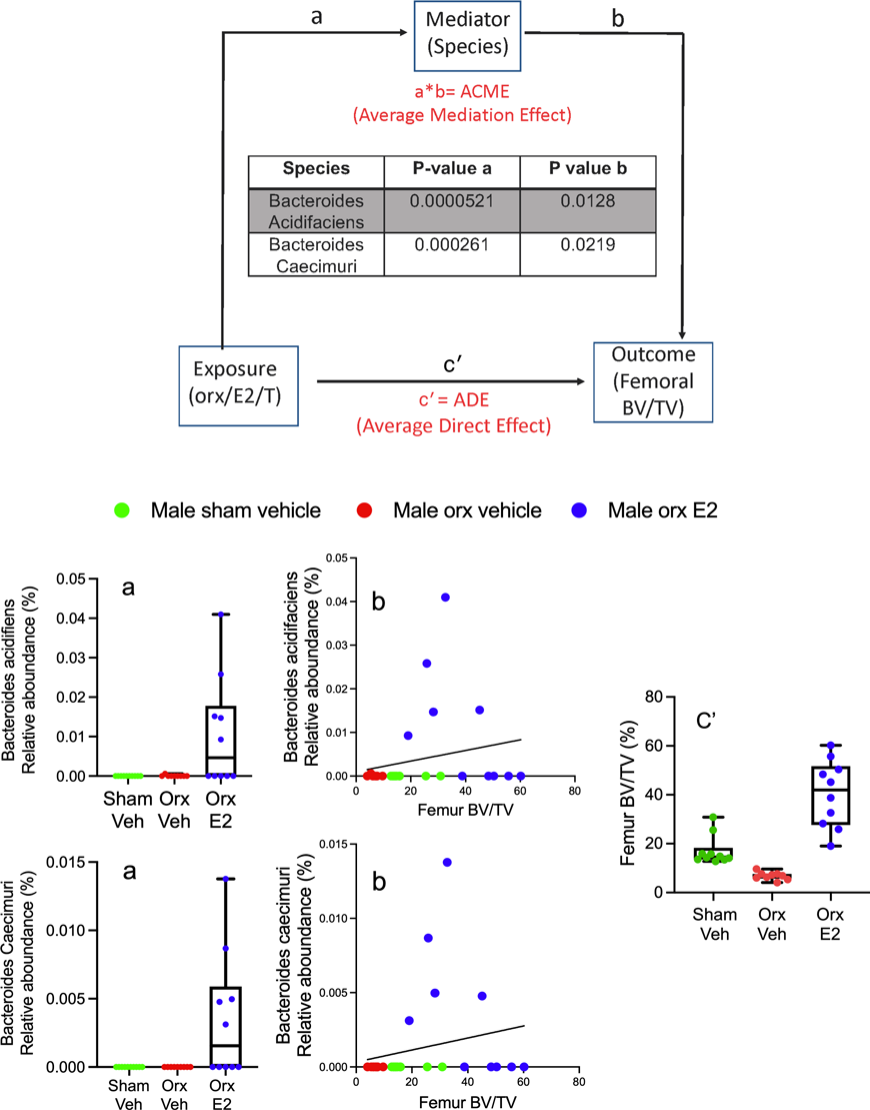
Mediation analysis conducted to determine the existence of a cause-effect relationship between orx or hormone treatment (exposure), bacterial species (mediators), and femoral BV/TV (outcome). This analysis identified 2 *Bacteroides* species that act as significant mediators (table within top panel). The bottom panels show graphic representations of a,b and c′ for *Bacteroides acidifaciens* and *Bacteroides caecimuri*. Data in a and c′ are shown as median with interquartile range. The bars show the minimum and maximum values. The curve in b shows the simple linear regression line.

To acquire information on the taxa that mediated the skeletal effects of GAHT in male mice, a mediation analysis (40) was performed using the data from the metagenomics analysis of stools collected after 4 weeks of GAHT. This analysis assessed whether an exposure (orx or hormone treatment) affected an outcome (BV/TV) through a mediator (bacterial species or taxa). We found 2 species of *Bacteroides* (*Bacteroides acidifaciens* and *Bacteroides caecimuri*) to be significant mediators of BV/TV in male mice (Figure 6). As expected, no species were found to mediate the effects of GAHT on BV/TV in female mice. These findings suggest a causal role for GAHT-induced microbiome compositional changes, and, specifically, alterations in *Bacteroides* abundance, in modifying bone volume in male but not in female mice.

To investigate the role of *Bacteroides* as mediators of BV/TV in male mice we correlated femoral and spinal BV/TV with the relative frequencies of *Bacteroides acidifaciens* and *Bacteroides caecimuri* in the stools of ovx-vehicle recipient mice with microbiome from either sham-vehicle, orx-vehicle or orx-E2 donors. We found significant correlations between each of these 2 species of *Bacteroides* and spinal and femoral BV/TV (Supplemental Figure 15).

**Figure 7:**
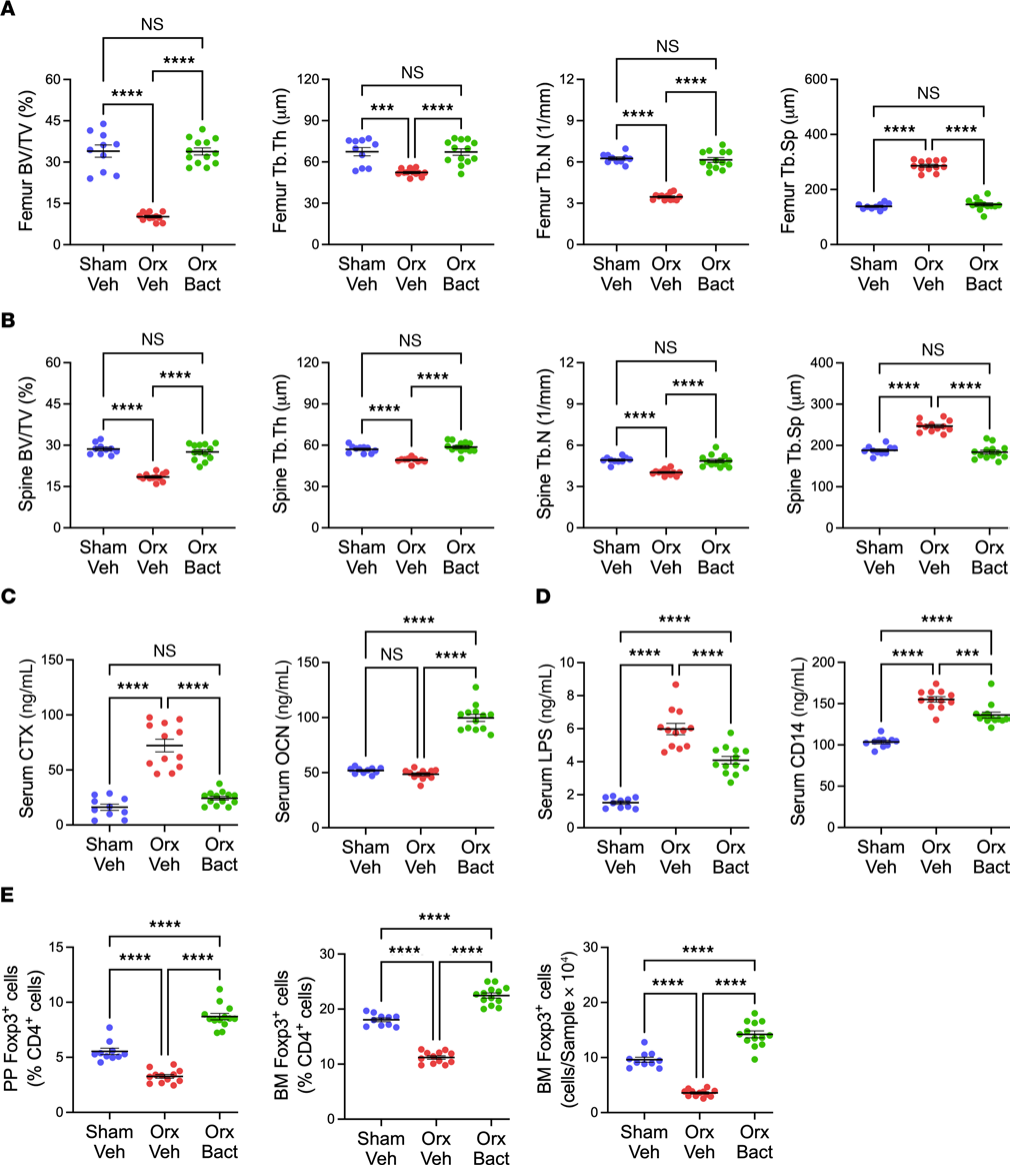
Effects of *Bacteroides* supplementation in male mice. 10-week-old orx or sham-orx mice were gavaged 3 times per week for 4 weeks with a liquid suspension of vehicle or *Bacteroides acidifaciens* JCM 10556 and *Bacteroides caecimuri* (1 × 10^6^ CFU of each strain), and then sacrificed. (**A**) Micro-CT indices of femoral trabecular bone structure. (**B**) Micro-CT indices of spinal (L4) trabecular bone structure. (**C**) Serum levels of the bone turnover indices CTX and OCN. (**D**) Serum levels of the gut permeability indices LPS and sCD14. (**E**) Relative and/or absolute frequency of PP and BM Treg (CD3^+^CD4^+^FoxP3^+^) cells. All data were normally distributed and were analyzed by 1-way ANOVA and post hoc tests applying Bonferroni’s correction for multiple comparisons. *n* = 10–13 mice/group. Data were expressed as Mean + SEM. ***P < 0.001, and ****P < 0.0001. Veh, vehicle. Bact, *Bacteriodes*.

To directly determine whether *Bacteroides* mitigate the bone loss induced by male sex steroid deprivation, 10-week-old male mice were orx and gavaged 3 times per week for the first 4 weeks after surgery with a liquid suspension of a mixture of *Bacteroides acidifaciens* JCM 10556 and *Bacteroides caecimuri* (1 × 10^6^ CFU of each strain). Vehicle-treated orx mice and vehicle-treated sham-orx were used as controls. Micro-CT measurements of bone samples harvested at sacrifice revealed that feeding of *Bacteroides* strains completely prevented the loss of BV/TV and the changes in Tb, Th, Tb.N, and Tb.Sp induced by orx, whereas feeding with vehicle did not (Figure 7, A and B). In addition, feeding of *Bacteroides* completely prevented changes in CTX, OCN, and indices of gut permeability induced by orx, whereas feeding with vehicle did not (Figure 7, C and D). These findings demonstrate that *Bacteroides* supplementation was as effective as E2 in preventing the changes in bone structure, bone turnover, and gut permeability induced by orx.

**Figure 8:**
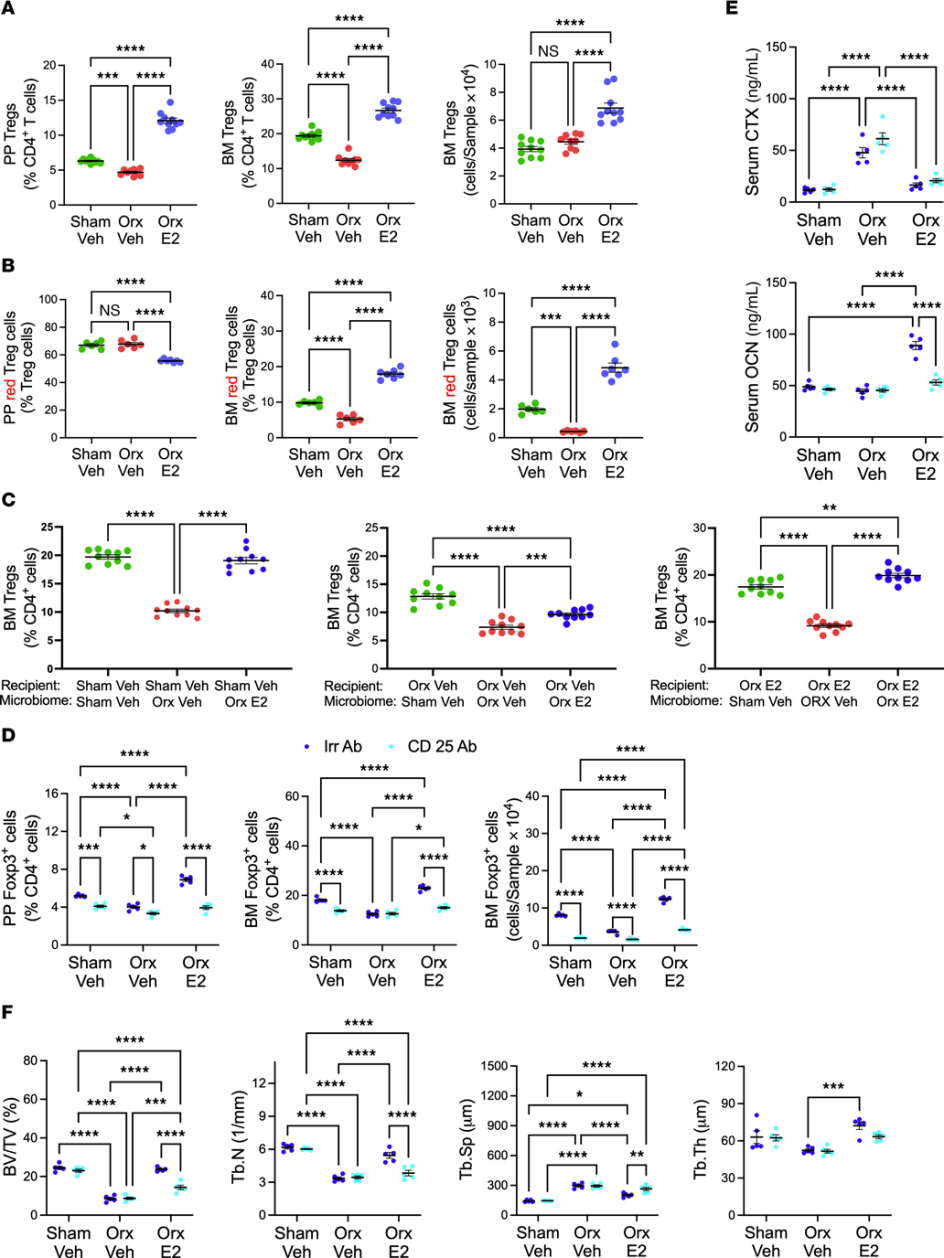
Effects of GAHT on PP and BM Tregs in donor and recipient mice, on the trafficking of PP and BM Tregs and effects of Treg depletion by anti CD25 Ab in male mice. (**A**) Relative and/or absolute frequency of PP and BM Treg (CD3^+^CD4^+^FoxP3^+^) cells in GAHT-treated donor male mice. (**B**) Relative frequency and absolute number of PP and BM red fluorescent tagged Treg cells in GAHT-treated male mice. (**C**) Relative frequency of BM Tregs in male recipient mice. (**D**). Effects of anti-CD25 Ab on the frequency of PP and BM Tregs in male GAHT-treated mice (**E**). Effects of anti-CD25 Ab on serum CTX and OCN in male GAHT-treated mice. (**F**). Effects of anti-CD25 Ab on micro-CT indices of trabecular structure in male GAHT-treated mice. *n* = 5–10 mice/group. All data were normally distributed and were analyzed by 1-way ANOVA (Panels **A**–**C**) or 2-way ANOVA (Panels **D**–**F**) and post hoc tests applying Bonferroni’s correction for multiple comparisons. Data were expressed as Mean + SEM. *P < 0.05, **P < 0.01, ***P < 0.001, and ****P < 0.0001. Nonsignificant comparisons not shown. Veh, vehicle.

*Bacteroides* are known to induce the expansion of Tregs (41–43), a cell lineage capable of suppressing osteoclastogenesis (29) and stimulating bone formation (27, 28). We analyzed by flow cytometry the frequency of BM and intestinal Peyer’s patches (PPs). Because the measurement of the absolute number of PP cells is technically challenging due to variability of the size of the collected PP tissue, PP Tregs were quantified as percentage of CD4^+^ T cells. Analysis of male mice supplemented with *Bacteroides* revealed that orx-vehicle mice had a lower frequency of PP and BM Tregs compared with the other 2 groups (Figure 7E). Moreover, orx *Bacteroides*-supplemented mice had a higher frequency of PP and BM Tregs than sham-vehicle and orx-vehicle groups (Figure 7E). Analysis of male mice treated with GAHT revealed that the frequency of PP and BM Tregs in orx E2-treated mice was higher than in sham-orx controls and orx vehicle-treated mice (Figure 8A), demonstrating that estrogen is more potent than endogenous testosterone in expanding intestinal and BM Tregs. The finding that testosterone deficiency affected the size of the pool of PP and BM Tregs extends the previously described effects of estrogen withdrawal on Tregs in female mice (21, 44). Studies in eugonadic donor female mice revealed that testosterone treatment increased the abundance of PP Tregs but did not alter the number of BM Tregs (Supplemental Figure 16A), pointing to more potent effects of testosterone in expanding intestinal Tregs and to similar effects of endogenous estrogen and testosterone treatment in regulating BM Tregs.

BM Tregs are likely to originate from microbiome-induced intestinal Tregs. To directly investigate the effect of orx and E2 treatment on Treg trafficking, we made use of male SFB^+^ C57BL/6 Kaede mice (45). This strain offers a sensitive means to track the migration from the gut to anatomically distant sites of any leukocyte cell type definable by surface-displayed or intracellular markers. Kaede mice ubiquitously express the photoconvertible protein Kaede, which permanently changes its fluorescence emission from green (518 nm) to red (582 nm) upon photoactivation with near-UV light (350–410 nm). Once photoconverted in the intestine, red-fluorescing cells can be detected and enumerated by flow cytometry in other organs. The photoconversion of intracellular Kaede has no effect on cellular function and on the homing capacity of T cells (46). Hereafter, we will refer to photoconverted cells as KaedeR cells. Male 5-week-old Kaede mice were orx or sham operated. Starting 1 week after surgery, Kaede mice were then treated with E2 or control vehicle for 4 weeks. Thereafter, all animals were subjected to surgical laparotomy and 4 PPs/mouse were photoactivated. Mice were sacrificed 24 hours later and the number of KaedeR Tregs in PPs and BM were measured by flow cytometry. Analysis of the cells harvested from the photoactivated PPs revealed that 24 hours after photoactivation E2-treated orx mice had a lower relative frequency of PP KaedeR Tregs than the other 2 groups (Figure 8B), indicating that E2 increased the egress of Tregs from PPs. Analysis of BM cells revealed that vehicle-treated orx mice had lower relative and absolute frequency of BM KaedeR Tregs, while E2-treated orx mice had an increased frequency of BM Tregs compared with the other 2 groups (Figure 8B). Since only photoconverted intestinal cells fluoresce red, these data demonstrate that E2 promoted the migration of intestinal Tregs from intestinal tissues to the BM.

To determine if orx and E2 replacement affects intestinal Tregs via a microbiome-dependent mechanism, we analyzed PPs of FMT recipient mice. Sham-orx recipient male mice with orx-vehicle microbiomes had a lower frequency of PP and BM Tregs than those with microbiomes from the other 2 groups (Figure 8C, left panel). Orx vehicle-treated recipients with concordant microbiomes had a lower frequency of PP and BM Tregs than those with discordant microbiomes (Figure 8C, middle panel). Orx-E2 recipient male mice with concordant microbiomes had a higher frequency of PP and BM Tregs than those with discordant microbiomes (Figure 8C, right panel). In female mice, vehicle-treated recipient mice with concordant microbiomes had a higher frequency of PP Tregs than those with discordant microbiomes (Supplemental Figure 16B, left panel). Moreover, testosterone-treated recipient female mice with concordant microbiomes had a lower frequency of PP Tregs than those with discordant microbiomes (Supplemental Figure 16B, right panel). However, FMTs did not alter the relative frequency of BM Tregs in any group (Supplemental Figure 16C). Together, our findings suggest the existence of direct effects of GAHT-shaped microbiomes on BM and intestinal Tregs in male mice and intestinal Tregs in female mice.

Because CD25 antibody (Ab) depletes Tregs in vivo (27, 47), mice were treated with anti CD25 Ab or isotype matched irrelevant Ab (Irr. Ab) to investigate the contribution of Treg expansion to the skeletal effects of E2 treatment in male orx mice. In these experiments, male mice were orx or sham operated at the age of 5 weeks and treated with vehicle or E2 for 4 weeks, starting at 6 weeks of age. Mice were also treated with anti-CD25 Ab or isotype matched Irr. Ab twice a week for 5 weeks starting at surgery, as previously described (27, 28). The expansion of intestinal and BM Tregs induced by E2 treatment was blunted in mice injected with an anti-CD25 Ab, but not in those injected with Irr. Ab (Figure 8D). Analysis of serum markers of bone turnover revealed that anti-CD25Ab blocked the increase in OCN induced by E2 treatment (Figure 8E). By contrast, anti CD25 Ab did not affect the increase in CTX induced by orx, suggesting that Tregs are implicated in the stimulation of bone formation induced by E2, but not in the increase in bone resorption induced by orx. In vitro micro-CT analysis of femurs harvested at sacrifice revealed that anti-CD25 Ab treatment prevented the increase in BV/TV and the changes in Tb.N and Tb.Sp induced by E2 treatment in orx mice (Figure 8F), demonstrating the functional role of Tregs as mediators of the bone anabolic effects of E2 in orx mice.

## Discussion

We report that in young male and female mice, GAHT did not impair skeletal maturation. Indeed, in male mice, estrogen supplementation resulted in a net improvement in bone structure driven by increased bone formation. We show that in male and female mice, GAHT altered the composition of the gut microbiome and induced changes in bacterial metabolic pathways in a sex-specific manner. In male mice, GAHT-induced modifications to the microbiome were mechanistically relevant because replacement of the original and established gut microbiome by a new E2-shaped microbiome resulted in the acquisition by the recipient mouse of some of the skeletal features of the donor mouse. Mediation analysis identified *Bacteroides* as a taxa contributing to the skeletal effects of GAHT in male mice. In keeping with the capacity of *Bacteroides* to induce Treg differentiation (48, 49), GAHT altered the frequency of BM Tregs in male mice, providing a potential mechanism whereby GAHT-shaped microbiomes regulated bone structure and bone formation. By contrast, in female mice, GAHT-induced modification of gut microbiome composition did not lead to improved skeletal maturation. The reason for the sexually dimorphic contribution of the gut microbiome to the skeletal activity of GAHT remains unknown, although testosterone treatment of female mice did not induce the expansion of bacterial species capable of altering Tregs or other T cell populations with bone-regulating activities.

Studies have highlighted sex as a factor affecting the composition of the human gut microbiota (14, 15). Differences in the gut microbial composition have been reported to be driven by gonadal steroids (18), particularly testosterone (50), with menopause also affecting gut microbiome composition (50). Animal studies confirmed significant differences in gut microbiota composition according to sex (16, 17). Castration in male mice abolished sex-dependent differences in gut microbiota composition, suggesting a role for testosterone in upholding diversity in the microbiome of eugonadic males (51). Our data not only corroborated this report, but also provided evidence that although sex affected the composition of the gut microbiome at the species level, it did not alter functional aspects of the microbiome, such as metabolic pathways.

Human and animal studies have implicated the gut microbiome as a regulator of BMD (20, 21), bone tissue material properties (52), bone mineral absorption (53), bone metastasis (54), and the pathogenesis of osteoporosis (55). Studies have shown differences in microbiome diversity and composition between individuals with and without osteoporosis (55). By utilizing maternal/offspring transmission, cohabitation, or FMT studies, we reported that the gut microbiome is a communicable regulator of bone maturation and postnatal skeletal development in mice (22). Moreover, the gut microbiome was found to act as a major nongenomic heritable factor in the transmission of bone phenotypes, including from mother to offspring (22). In the current study, FMTs were carried out to determine if skeletal phenotypic features of GAHT-treated mice were transmissible to recipient mice. Although stools may contain low levels of conjugated and unconjugated sex steroids (56), we found undetectable levels of E2 in the stool of donor mice. We also found that transfer of microbiomes from E2-treated orx donors did not increase serum levels of E2 in any group of recipient mice. Together, these findings argue against the possibility that transfer of fecal E2 might have altered the overall level of estrogen in recipient mice and contribute to bone anabolism. Therefore, the findings of the current study demonstrated the relevance of gut microbiome for skeletal maturation of male mice treated with GAHT. Moreover, while it is now established that the presence of the microbiota is required for estrogen deficiency to cause bone loss (21), the finding that the transfer of stools from E2-treated mice into orx recipients attenuates orx-induced bone loss provided evidence that the microbiome mediates the bone loss induced by orx.

Previous investigations had revealed a mechanistic link between the gut microbiome, gut permeability, and bone loss induced by estrogen deficiency (21, 30, 44). The current study revealed that, like estrogen deficiency, testosterone deficiency induced by orx increased gut permeability by downregulating claudins and Jam3 proteins. Moreover, we found that estrogen replacement in orx mice normalized gut permeability, while testosterone treatment was equally effective as endogenous estrogen in maintaining low permeability in female mice. These data suggest that estrogen and testosterone are both potent regulators of gut permeability. We hypothesize that regulation of gut permeability is likely to be a key mechanism by which sex steroids promote skeletal development and maintain skeletal health. Firstly, increased gut permeability induces dysbiosis (57), which further increases permeability. Secondly, increased gut permeability decreases intestinal Tregs (58), while Tregs improve gut barrier function (59). Thirdly, increased gut permeability leads to microbial antigen translocation across the intestinal barrier (60). Microbial components (e.g., LPS) in concert with specific Th17 cell-inducing bacteria such as SFB (61) expand intestinal Th17 cells (21, 39). These cells stimulate bone resorption and blunt bone formation via cytokine secretion. Thus, several mechanisms link increased permeability to bone loss. Therefore, our findings provide support to the hypothesis that GAHT regulates skeletal development both by preserving gut barrier integrity and by inducing positive changes in the gut microbiome.

Our data revealed that, in male mice, GAHT shaped microbiomes induced the expansion of intestinal and BM Tregs. Evidence of the role of Tregs as mediators of the effects of the microbiome on bone includes blockade of Treg expansion by anti-CD25 Ab treatment prevented the bone anabolic activity of E2. Moreover, we found *Bacteroides* supplementation to promote intestinal and BM Treg expansion and to mimic GAHT-induced bone anabolism. Tregs are suppressive CD4^+^ T cells critical for intestinal health (62). Tregs are induced by estrogen (25) and by testosterone (26), repress osteoclastogenesis (29), and stimulate bone formation by promoting the release of Wnt10b by CD8^+^ T cells, leading to Wnt signaling activation in osteoblastic cells (27, 28). Attesting to the relevance of Tregs for bone anabolism, Tregs are required for PTH, butyrate, and certain probiotics to stimulate bone formation (27, 28). In addition, Tregs have been implicated in mitigating the bone loss induced by ovariectomy and arthritis (24, 63). Studies have hypothesized that the BM Tregs relevant for bone health originate in the gut. To directly investigate whether orx and E2 treatment regulate the trafficking of Tregs from the gut to the BM, we made use of C57BL/6 Kaede mice (45). We previously used Kaede mice to demonstrate that ovariectomy increases the migration of TNF^+^ T cells and Th17 cells from the gut to the BM (44), and to establish that tumor bone growth attracts intestinal NK and Th1 cells to the tumor site (54). Other investigators have used Kaede and similar mice strains to track the migration of intestinal immune cells, including Th17 cells, to the kidney (64) and mesenteric lymph nodes (65).

In this study, we analyzed functional features of the gut microbiome by using the MetaCyc pathways database (38). The MetaCyc database (MetaCyc.org) is a comprehensive database describing more than 2,400 metabolic pathways from all domains of life. The majority of MetaCyc pathways are small-molecule metabolic pathways that have been experimentally determined. We did not attempt to identify specific pathways that may contribute to the effects of GAHT. We rather utilized functional profiling to pinpoint differences in the community of bacteria that established in response to GAHT.

In summary, we found GAHT not to impair skeletal maturation in young female mice and to improve bone structure via increased bone formation in male mice. In male mice, GAHT acted, in part, by regulating microbiome composition and function and gut permeability. Mechanistically, GAHT-induced microbiome changes may regulate bone maturation via Treg differentiation. The findings of this study may be relevant to human medicine as they suggest that initiation of GAHT therapy in TG adolescents is unlikely to impair skeletal maturation.

## Methods

### Sex as a biological variable.

Our study examined male and female animals and sexually dimorphic effects are reported.

### Mice.

Four-week-old SFB^+^ C57BL/6NTac mice were purchased from Taconic Biosciences. All mice originated from the same vivarium at Taconic Biosciences. Upon arrival at Emory University, the presence of SFB in the fecal sample of all mice was confirmed by qPCR (66). At 4 weeks of age, mice were randomly assigned to experimental groups in cages containing 5 mice each. Between 4 and 5 weeks of age, the microbiome of experimental animals was equalized by daily exchange of bedding between cages. All mice were housed in the same room of the same vivarium under specific pathogen–free clean conditions and were fed γ-irradiated 5V5R mouse chow (Purina Mills) and autoclaved water ad libitum. The animal facility was kept at 23°C (± 1°C) with 50% relative humidity and a 12-hour light/12-hour dark cycle.

### Orchiectomy and sham orchiectomy.

Mice were anesthetized by isoflurane 2.5%. Surgery was performed during the “surgical plane of anesthesia” as determined by the animal’s breathing and lack or response to the toe pinch. Mice were shaved and cleansed 3 times with alternating betadine and alcohol scrubs. Eyes were protected with ophthalmic ointment. An incision measuring 0.5–0.75 cm was made in the midline of the scrotum using a size 15 scalpel blade. The testicular fat pad was pulled to cut the cremaster muscle, and the testicle was pulled through the incision. The testicular fat pad was dissected to expose the cauda epididymis, caput epididymis, vas deferens and the blood vessels. While holding the testicle with toothed forceps, the aforementioned tissues were cauterized. The remaining stump was examined for bleeding before being returned to the scrotal sack. This process was repeated for the remaining testicle. In sham operated mice, the testicles were left intact without removal. The skin was closed with surgical sutures.

### GAHT.

To model male-to-female transition, male mice were orx at 5-week of age. Orx mice were treated with weekly subcutaneous injections of either vehicle or 17β estradiol E2 benzoate dissolved in 100 μl sesame oil, which is the dose used in humans allometrically adjusted to the average adult C57BL/6 bodyweight (6.4 μg/wk), for 10 weeks, starting 1 week after orx or sham surgery (11). Control mice were subjected to sham-orx and treated with weekly subcutaneous injections of vehicle. To model female-to-male transition, intact female mice were treated with weekly subcutaneous injections of testosterone in the allometrically weight-adjusted amount used for humans undergoing GAHT (31 μg/wk dissolved in 100 μl sesame oil) (11). Control female mice were injected with vehicle. At the endpoint, uterine weight was measured to evaluate the effect of estradiol level suppression in testosterone-treated animals.

### Bacteroides administration.

Six-week-old SFB^+^ C57BL/6NTac male mice were orx or sham operated and gavaged daily for 4 weeks with 1 mL vehicle or a liquid suspension of cultures of *Bacteroides acidifaciens* JCM 10556 (DSM No. 15896) and *Bacteroides caecimuris* (DSM No. 26085) (1 × 10^6^ CFU of each strain) purchased from DSMZ-German Collection of Microorganisms, Germany. Mice were then sacrificed and samples analyzed.

### Anti-CD25 Ab treatment.

Treg depletion was carried out by treating mice with anti-CD25 Ab as previously described (27, 28). Briefly, anti-CD25 Ab (clone PC61, 500 μg/mouse/injection IP) or isotype matched irrelevant Ab (BioXCell) were administered in GAHT-receiving mice twice a week for 5 weeks starting a week before beginning GAHT. All mice were sacrificed at the age of 10 weeks as endpoint.

### FMT experiments.

SFB^+^ C57BL/6NTac mice were used as microbiome donor and recipient mice in all FMT experiments. Stool samples from each donor were gavaged to a single recipient mouse. Each group of donor mice (9–10 mice pre group) was housed in 2 cages. Stool samples from each donor group were collected and pooled. Stool suspensions were prepared and gavaged in 9–10 recipient mice per group. Groups of microbiome recipient mice were prepared as follows: Male and female mice were recruited at 4 weeks of age and treated for 1 week with broad spectrum antibiotics (1 mg/mL ampicillin, 0.5 mg/mL vancomycin, 1 mg/mL neomycin sulfate, and 1 mg/mL metronidazole [all from Sigma-Aldrich]) to deplete the gut microbiome. Male mice were orx or Sham-orx surgery at 5 weeks of age. Female mice were not subjected to surgery. Starting at 5 weeks of age, male and female recipient mice were gavaged with liquid suspensions of stool material collected from donor mice. Gavages were performed 3 times during the first week, and once-a-week thereafter, until the completion of the experiment at 16 weeks of age. From 6 to 16 weeks of age, orx mice were treated with vehicle or E2. Sham-orx mice were treated with vehicle. Intact female mice were treated with vehicle or testosterone. FMT experiments were set up using a 3 × 3 design for male mice and a 2 × 2 design for female mice so that each group of recipient mice was gavaged with stool samples from each group of gender matched donors. The 9 treatment groups for male recipient mice study were as follows: sham vehicle mice + microbiome from sham vehicle donors; sham vehicle mice + microbiome from orx vehicle donors; sham vehicle mice + microbiome from orx-E2 donors; orx vehicle mice + microbiome from sham vehicle donors; orx vehicle mice + microbiome from orx vehicle donors; orx vehicle mice + microbiome from orx-E2 donors; orx-E2 mice + microbiome from sham vehicle donors; orx-E2 mice + microbiome from orx vehicle donors; and orx-E2 mice + microbiome from orx-E2 donors. The 4 treatment groups for male recipient mice study were as follows: vehicle-treated female mice + microbiome from vehicle-treated donors; vehicle-treated female mice + microbiome from testosterone-treated donors; testosterone-treated female mice + microbiome from vehicle-treated donors; and testosterone-treated female mice + microbiome from testosterone-treated donors.

### Kaede mouse cell trafficking study.

SFB^–^ Kaede mice [B6.Cg-c/c Tg(CAG-tdKaede)15Utr] were purchased from RIKEN Bioresource Research Center. SFB^+^ SFB^+^ Kaede mice were generated by oral gavaging SFB- mice with a liquid suspension of fecal pellets collected from SFB mono-associated mice, as previously described (44, 54). Kaede mice express a photoconvertible fluorescence protein that changes from green (518 nm) to red (582 nm) upon exposure to near-UV (350–410 nm) light. Studies were conducted as previously described (44, 54). Briefly, 5-week-old male C57BL/6 Kaede mice were subjected to orx or sham operation at 5 weeks of age and treated with vehicle or E2 for 4 weeks starting at 6 weeks of age. At 10 weeks of age, all mice underwent laparotomy during which the caecum and distal SI were eviscerated and the 4 PPs most proximal to the cecum were identified and illuminated with 390 nm wavelength light for 2 minutes each, to photoconvert them from green to red. Aluminum foil was used to protect tissues other than target PPs from light during exposure. The caecum and distal SI were reinserted in the abdominal cavity and the abdominal wall was closed. Mice were sacrificed 24 hours after photoconversion, and PP and BM cells were collected, and single cell suspension prepared. Red fluorescing Tregs were enumerated by flow cytometry in PP and BM.

### Micro-CT measurements.

Micro-CT scanning and analysis of the distal femur and spine were performed as reported previously (44, 54) using a Scanco micro-CT 50 scanner (Scanco Medical). Femoral trabecular bone regions were evaluated using isotropic 12 μm voxels. For the femoral trabecular region, we analyzed 70 slices starting 20 slices below the distal growth plate. For the spinal trabecular region, contours along the endosteal surfaces were drawn encompassing 100 slices of the L4 vertebra, starting at the beginning of trabecular bone within the spinal body. X-ray tube potential was 70 kVp, and integration time was 200 ms. We used the thresholding approach described by Bouxsein et al. (67), which is recommended by the manufacturer, and involves a visual inspection and comparison of preview and slice-wise grayscale 2D images. The same threshold value was used for all measurements.

### Bone turnover marker measurement.

Serum CTX (Immunodiagnostic Systems) and OCN (Quidel Corporation) were measured by rodent-specific ELISA assays.

### Preparation of PP and BM single cell suspension.

For PP cell isolation, the small intestine was removed and flushed of fecal content. PPs were excised and collected in 1 mL cooled RPMI 1640 (Corning). PPs were dissociated using the plunger of a 2.5 mL syringe (Bd Bioscience) and gently forced through a 70 μm cell strainer (Corning) placed over a 50 mL tube (Corning). A single cell suspension was used for flow cytometric analysis. For bone marrow (BM) cell isolation, femur, tibia, and pelvic bones were flushed with PBS (Corning) and BM cells were collected. RBC lysis was performed twice to eliminate all the RBCs from BM. Single cell suspension of BM cells was used for analysis by flow cytometry, as previously described (39, 44).

### Flow cytometry.

Flow cytometry was performed on a FACS Cytek Aurora (Cytek Bioscience), and data were analyzed using FlowJo software (Tree Star Inc.). For cell-surface staining, cells were stained with anti-mouse purified CD16/32 (Fc blocking Ab, clone 93, catalog 101302), BV 510-CD45 (clone 30-F11, catalog 103138), BV 421-TCR-β (clone H57-597, catalog 109230), Alexa488-CD3 (clone 17A2, catalog 100210), PerCP/Cy5.5-CD4 (clone RM4-5, catalog 100540), BV 711-CD8 (clone 53-6.7, catalog 100748) (all from BioLegend), The live cells were discriminated by the LIVE/DEAD Fixable Yellow Dead Cell Stain Kit (Thermo Fisher Scientific). For intracellular staining, cells were incubated with cell activation cocktail (BioLegend) in the presence of Monensin Solution at 37°C for 12 hours. Anti-mouse APC-FOXP3 (clone FJK-16s, catalog 17-5773-82) (Thermo Fisher Scientific) was added after cell fixation and permeabilization with Intracellular Fixation & Permeabilization Buffer Set (Thermo Fisher Scientific).

### E2 level measurement.

Serum 17β-estradiol level was measured at endpoint by using rodent-specific ELISA (R&D Systems) following manufacturer protocol.

### Gut barrier function evaluation.

Gut barrier function was evaluated by measuring the serum level of LPS (Cusabio) and CD14 (R&D Systems) by rodent-specific ELISA assays.

### qPCR of intestinal tight junction proteins.

For the evaluation of gut gap junction protein expression, the mRNA levels of claudin 2, claudin 3, claudin 15, and Jam3 were measured in small intestine by qPCR. RNA was isolated using RNeasy kit (QIAGEN), and cDNA was synthesized using Superscript II (Invitrogen) and random hexamers according to the manufacturer’s instructions. Relative abundance of cDNAs was determined by qPCR analysis using the ABI StepOnePlus Real-Time PCR system (Applied Biosystems). All the primers used were designed by Primer Express Software v3.0.1 (Applied Biosystems) Changes in relative gene expression were calculated using the 2^–ΔΔCT^ method with normalization to 18S rRNA. The primers used were as follows: 18S rRNA, 5′-ATTCGAACGTCTGCCCTATCA-3′ (forward) and 5′-GTCACCCGTGGTCACCATG-3′ (reverse); Cldn2, 5′-TCTCAGCCCTGTTTTCTTTGG-3′ (forward) and 5′-GGCGAGCAGGAAAAGCAA-3′ (reverse); Cldn3, 5′-TCATCACGGCGCAGATCA-3′ (forward) and 5′-CTCTGCACCACGCAGTTCA-3′(reverse); Cldn15, 5′-GGCGGCATCTGTGTCTTCTC-3′ (forward) and 5′-TGGTGGCTGGTTCCTCCTT-3′ (reverse); and Jam3, 5′-CACTACAGCTGGTACCGCAATG-3′ (forward) and 5′-CTGGGATTGGCTCTGGAATC-3′ (reverse).

### Metagenomic shotgun microbiome sequencing and bioinformatics analysis.

Microbiome composition and function were determined by a metagenomic shotgun sequencing methodology developed by CosmosID Inc. Briefly, DNA from fecal samples were isolated using the QIAGEN DNeasy PowerSoil Pro Kit. DNA libraries were prepared using the Illumina Nextera XT library preparation kit and the library quantity assessed with Qubit (Thermo Fisher Scientific). Libraries were sequenced on an Illumina HiSeq platform 2 × 150 bp. The average sequencing depth was 4,948,936 reads per sample (range: 1,240,758–14,788,148). Taxonomic assignment and functional analysis were performed using the CosmosID bioinformatics platform. Taxonomy was assigned using the kmer-based algorithms and the GenBook database, which comprises over 150,000 genomes from bacteria, viruses, fungi, and protists. The principal software pipeline has been optimized for processing unmapped and unaligned raw sequence reads of lengths less than 100 bp. For functional assignment, sequencing reads were mapped to Uniref90 genes and assembled into MetaCyc pathways using CosmosID software.

### Statistics.

Micro-CT indices, markers of bone turnover, and indices of gut permeability were normally distributed according to the Shapiro-Wilk normality test. These data were analyzed by unpaired 2-tailed *t* tests or 1-way ANOVA and Bonferroni correction for multiple comparisons, as appropriate. Microbiome data were quantified and visualized in R using the vegan, dplyr, heatmap.2, and ggordiplots packages. For microbial species and MetaCyc pathways, α diversity was analyzed by calculating the Shannon diversity index. α diversity was compared between 2 groups by the Wilcoxon-rank-sum test or among 3 groups by the Kruskal-Wallis test. β diversity was compared between species and MetaCyc pathways using the Bray-Curtis dissimilarity metric, utilized for ordinating the study samples by nonmetric multidimensional scaling analysis plots, and tested in association with a covariate (e.g., sex and treatment) by the PERMANOVA test (68). The microbial relative abundance data were tested for association with a covariate or for mediation of a treatment on BV/TV at both the community (global) level and the species level by the Linear Decomposition Model (LDM) (68, 69). LDM was built on a linear model that regresses the microbial relative abundance data on covariates and based on permutation for inference. Using a specific linear model that regresses the microbial data on the treatment and the treatment-adjusted outcome, the LDM was employed for testing the mediation effect of a treatment on an outcome. The species -level associations or mediations were detected by controlling the FDR ≤ 0.1.

### Study approval.

All animal procedures were approved by the Institutional Animal Care and Use Committee of Emory University in compliance with all applicable federal regulations governing the protection of animals in research.

### Data availability.

Sequencing reads are deposited at SRA: Bioproject PRJNA1071073. All R code used in analysis and data set is available at: https://github.com/morganx/Pal_murine_bones (commit ID: 1443c91). All data supporting the graphs are provided in the Supporting Data Values file. Data are also available from the corresponding author upon request.

## Author contributions

S Pal, MNW, and RP designed the studies. S Pal, HYD, CAG, S Patil, AS, YJH, and XM performed the research and analyzed the animal data. S Pal, RMJ, MNW, and RP wrote the manuscript.

## Supplementary Material

Supplemental data

Supporting data values

## Version 1 03/26/2024

In-Press Preview

## Version 2 05/15/2024

Electronic publication
