# Supplementary material for: Gender-affirming hormone therapy preserves skeletal maturation in young mice via the gut microbiome
Source: J Clin Invest. 2024 Mar 26;134(10):e175410. doi: 10.1172/JCI175410 (PMC11093603; doi:10.1172/JCI175410)
Supplement: Supplemental data [file jci-134-175410-s082.pdf]

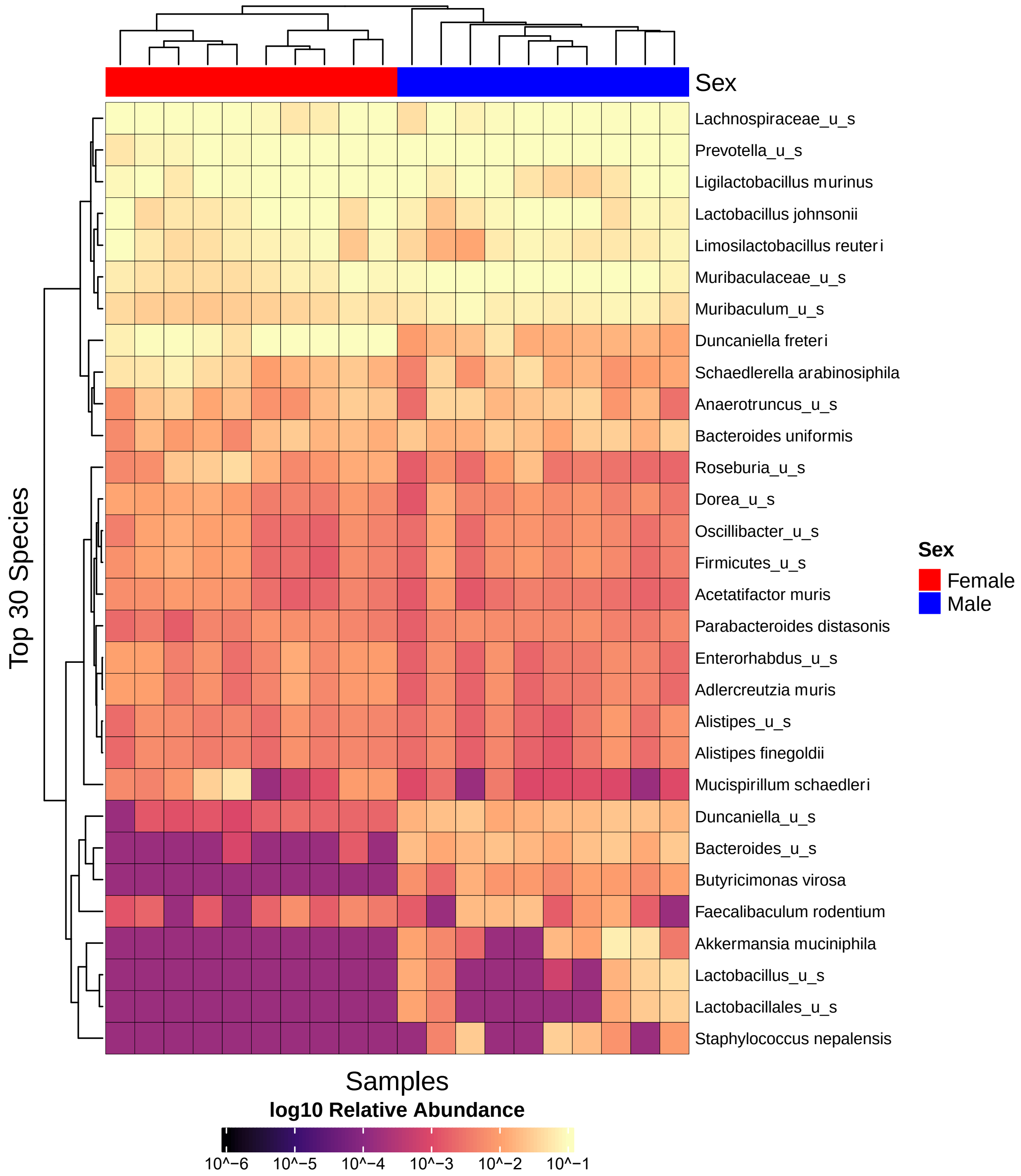

Supplemental Figure 1. Heatmap of bacterial species associated with sex in untreated mice. Stool samples were collected from 6-week-old male and female mice. Data were plotted for top 30 most abundant bacterial species in male and female mice.

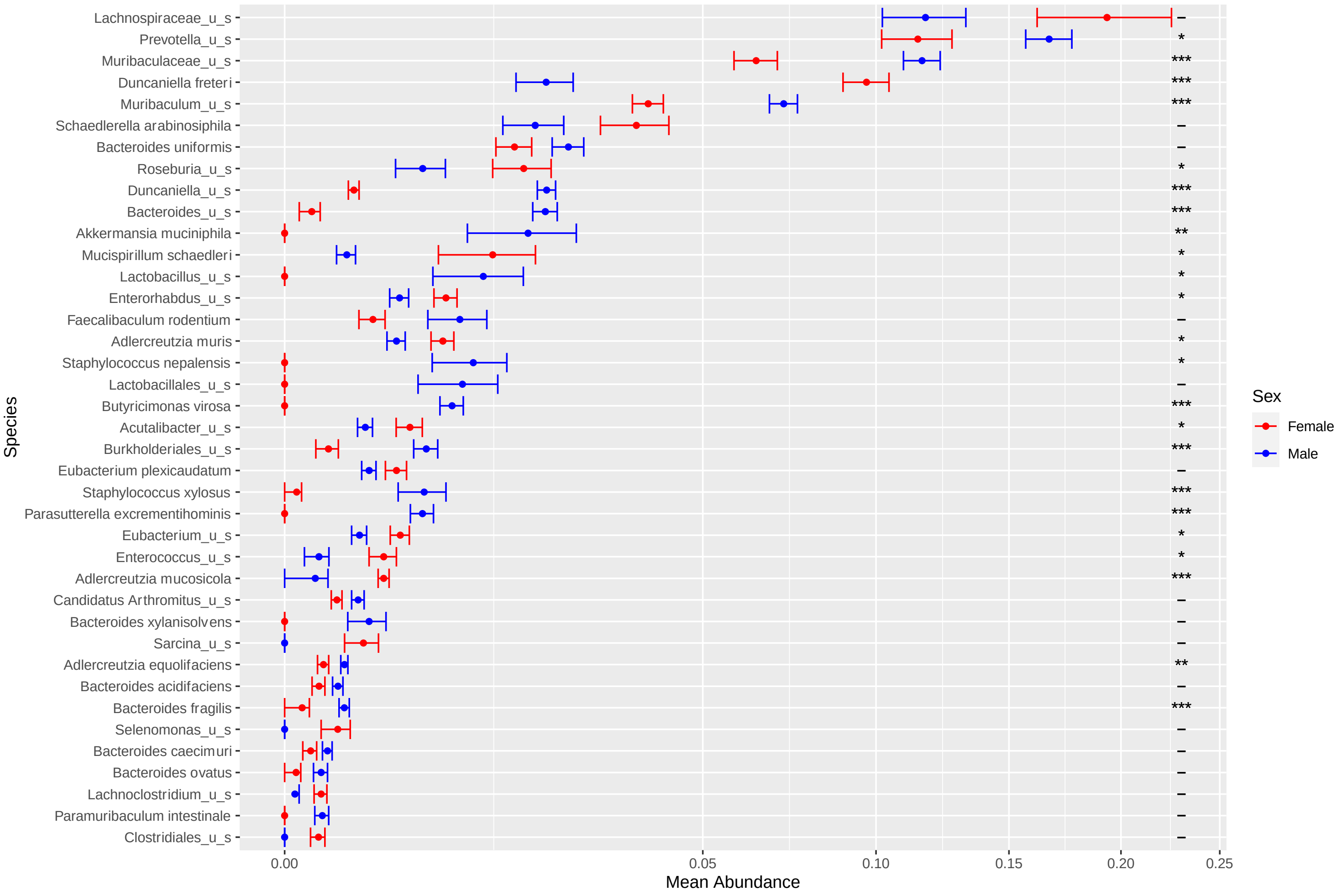

Supplemental Figure 2. Species associated with sex in untreated mice. Stool samples were collected from 6-week-old male and female mice. Data were plotted at the species level, comparing mean abundance of different bacterial species in male and female mice. Global P-value: 0.0002, FDR ≤ 0.1. Data were expressed as Mean ± SEM. * =p<0.05, ** =p<0.01, *** =p<0.001.

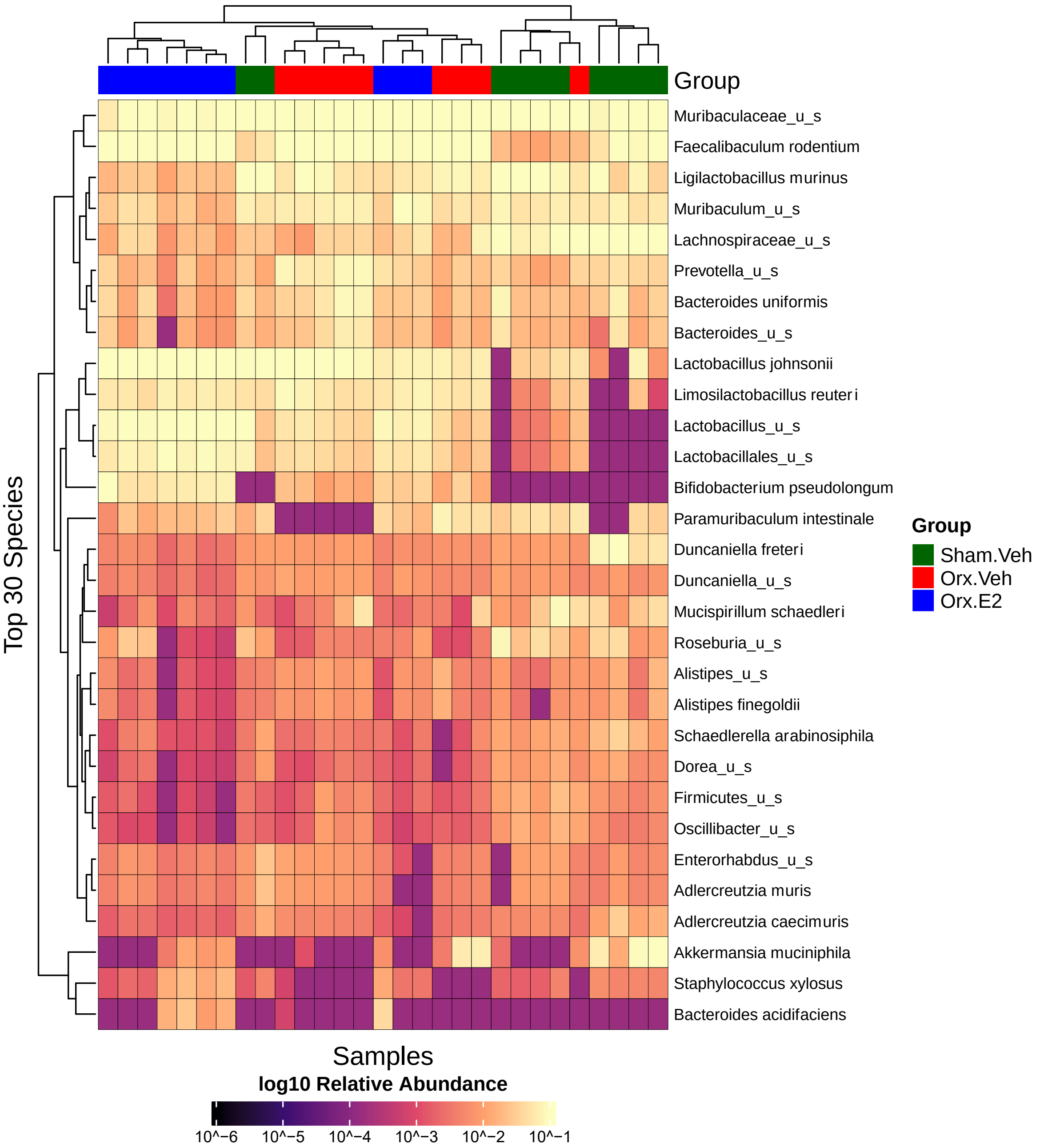

Supplemental Figure 3. Heatmap of species associated with treatment groups after 4 weeks of GAHT in male mice. Stool samples were collected from male mice treated with GAHT for 4 weeks. Data were plotted for top 30 most abundant bacterial species in male mice.

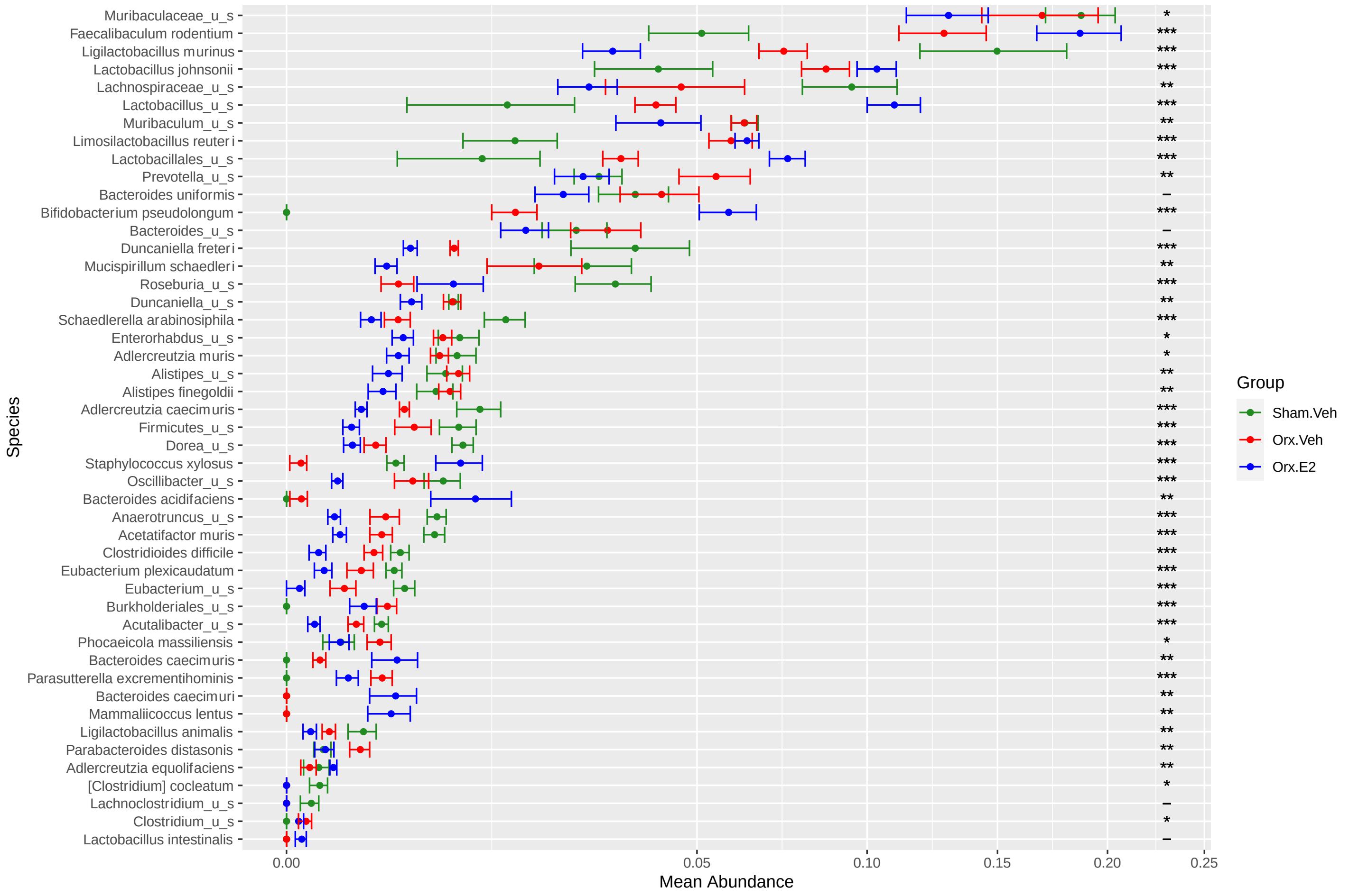

Supplemental Figure 4. Species associated with treatment groups after 4 weeks of GAHT in male mice. Stool samples were collected from GAHT treated male mice. Data were plotted at the species level, comparing mean abundance of different bacterial species in 3 treatment groups. Global P-value: 0.0002, FDR ≤ 0.1. Data were expressed as Mean ± SEM. * =p<0.05, ** =p<0.01, *** =p<0.001.

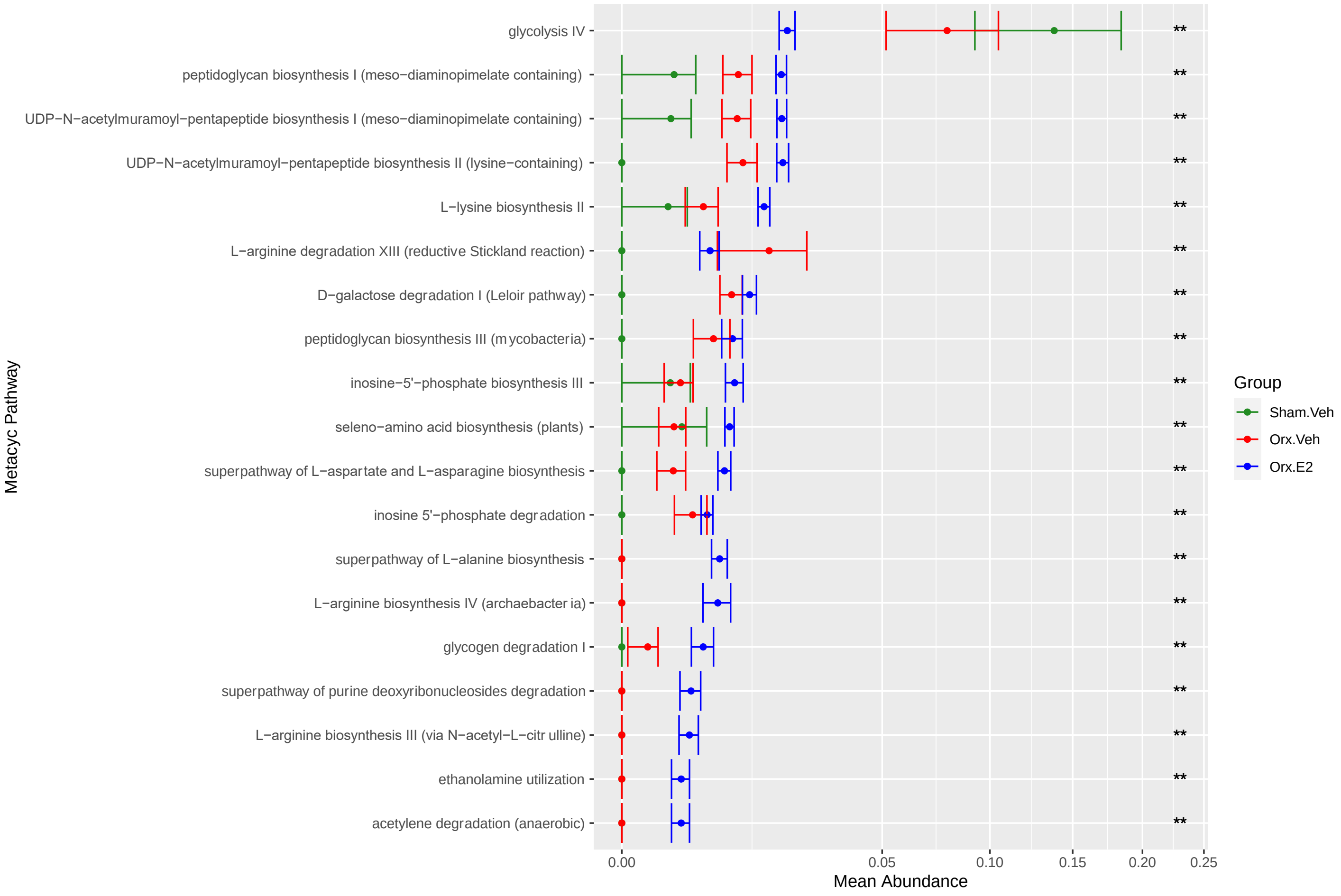

Supplemental Figure 5. Association between MetaCyc pathways and treatment groups in male mice. 49 MetaCyc pathways features were significantly different (Global P-value:0.0002, FDR ≤ 0.1) between vehicle treated sham mice, vehicle treated orx mice and E2 orx mice. The 19 metabolic pathways with the smallest p value are shown in the figure. Data were expressed as Mean ± SEM. ** = p<0.01.

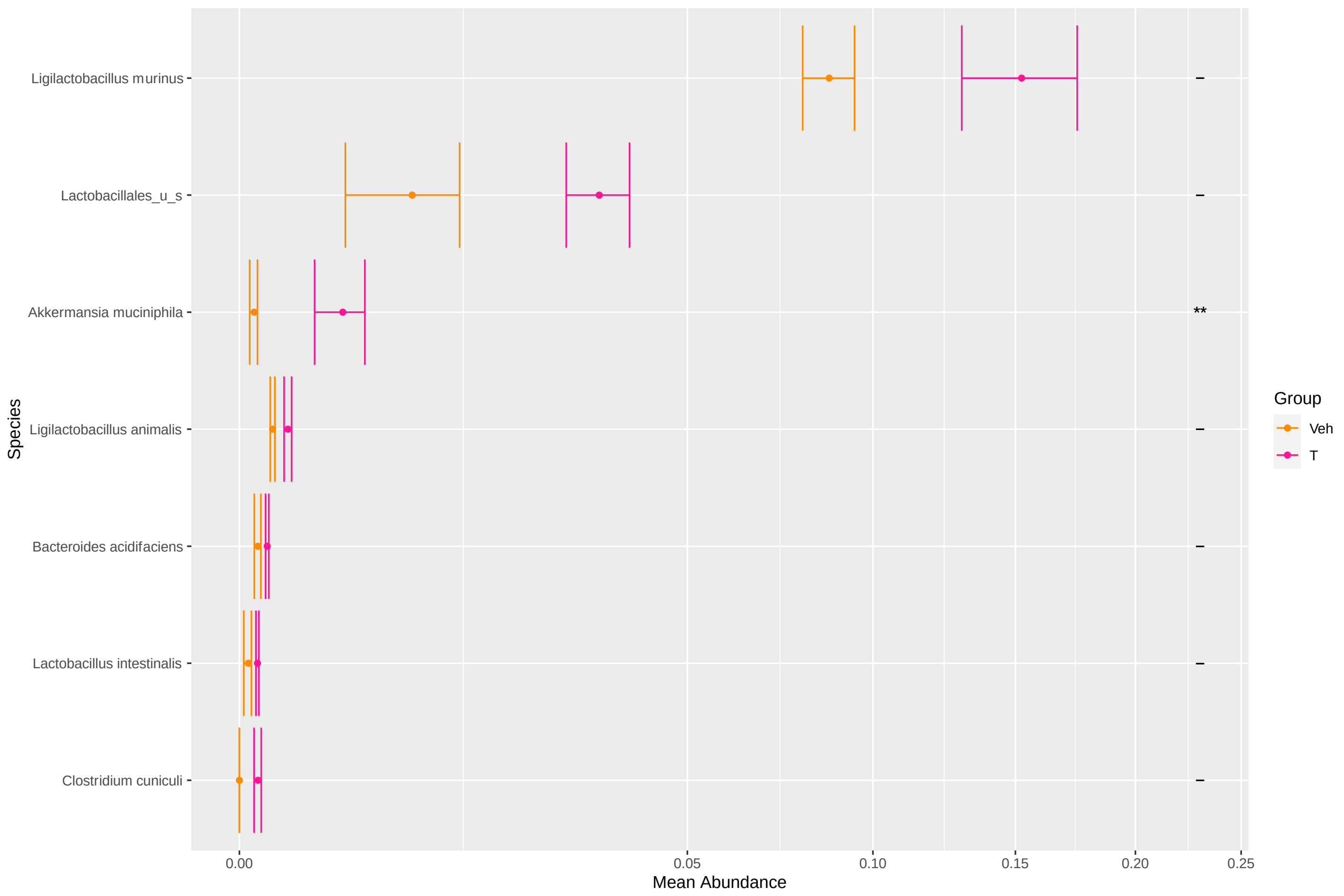

Supplemental Figure 6. Bacterial species associated with treatment groups after 4 weeks of GAHT in female mice. Stool samples were collected from GAHT treated female mice. Data were plotted at the species level, comparing mean abundance of different bacterial species in 2 treatment groups. Global P-value: 0.0006, FDR ≤ 0.1. Data were expressed as Mean $\pm$ SEM. ** = p<0.01.

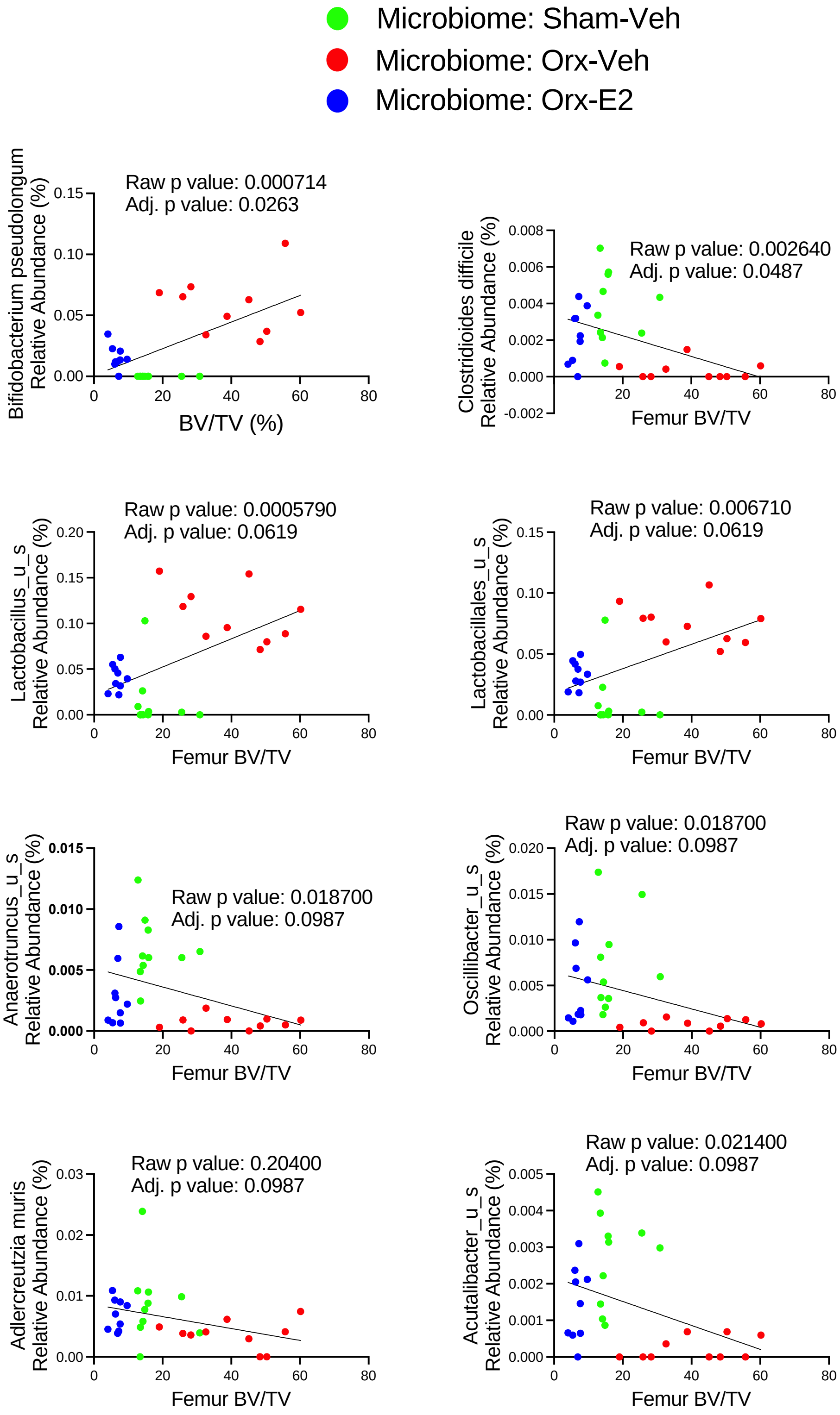

Supplementary Figure 7. Bacterial species associated with BV/TV in GAHT treated male mice. The figure shows the 8 species that were significantly associated with femoral BV/TV. Bacterial species relative abundance was tested for association with femoral BV/TV by the Linear Decomposition Model (LDM), which produced the species-level raw p-values and adjusted p-values by controlling the FDR ≤ 0.1, as well as a global p-value of 0.034 for testing any difference in the entire community. The line in each graph shows the fitting of data by simple linear regression. n= 29.

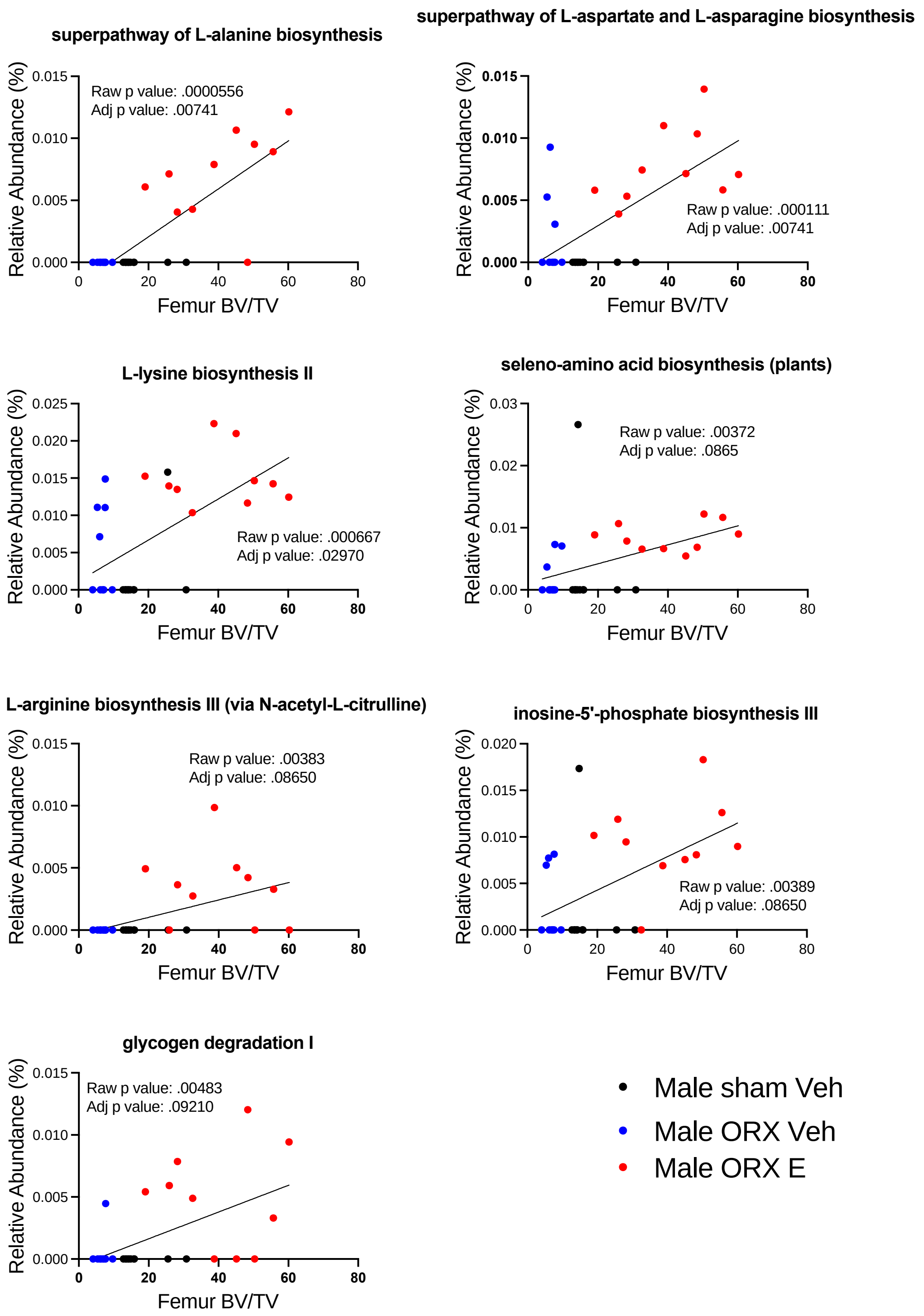

Supplementary Figure 8. Metabolic pathways associated with BV/TV in GAHT treated male mice. The figure shows the 7 metabolic pathways that were significantly associated with femoral BV/TV. Metabolic pathways relative abundance was tested for association with femoral BV/TV by the Linear Decomposition Model (LDM), which produced raw p-values and adjusted p-values by controlling the FDR ≤ 0.1, as well as a global p-value of 0.0465 for testing any difference in the entire community. The line in each graph shows the fitting of data by simple linear regression. n= 29.

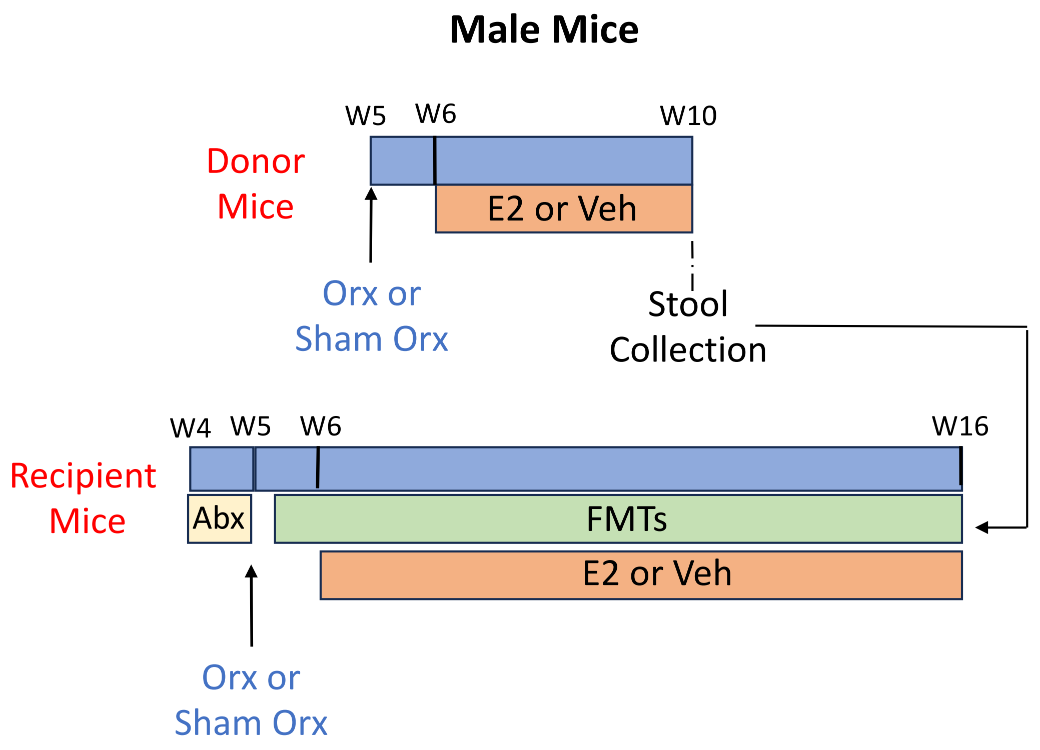

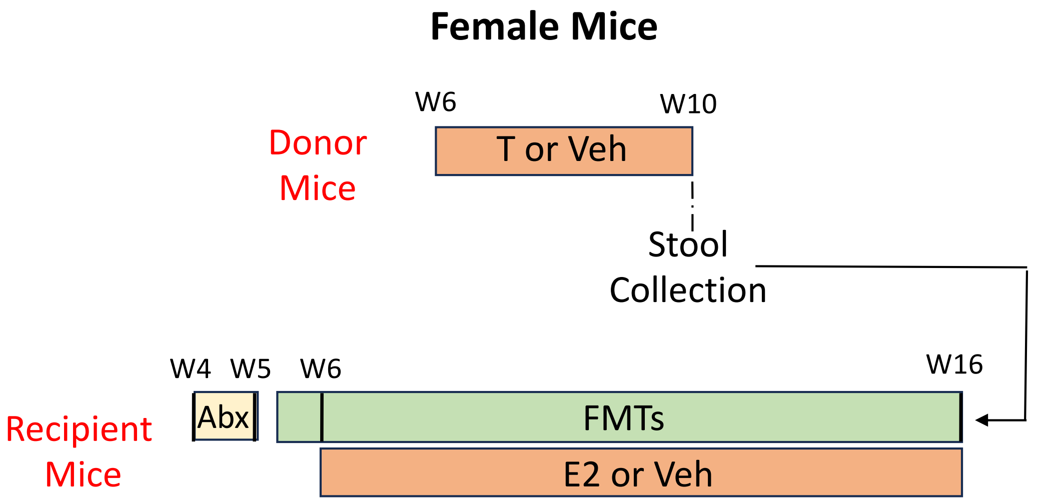

Supplemental Figure 9. Fecal microbiome transfer (FMT) experiment design. Male donor mice were sham-orx or orx at 5 weeks of age and treated with either vehicle or E2 for 4 weeks starting at 6 weeks of age. Intact female donor mice were treated with vehicle or testosterone for 4 weeks starting at 6 weeks of age. 4-week-old recipient male and female mice were treated with broad-spectrum antibiotics (Abx) for 1 week to deplete the microbiome. Recipient male mice sham-orx or orx at 5 weeks of age. After 1 week, sham-orx mice were started on vehicle, while the two groups of orx mice were treated with either vehicle or E2 for 10 weeks. Recipient female mice were treated with vehicle or testosterone for 10 weeks starting at 6 weeks of age. Two days after completion of the Abx treatment male and female recipient mice were subjected to FMTs by gavaging liquid suspensions of stool samples from donor mice for 10 weeks. FMTs were repeated 3 times during the first week and once a week thereafter to assure that the microbiome of donor mice was maintained in recipient mice.

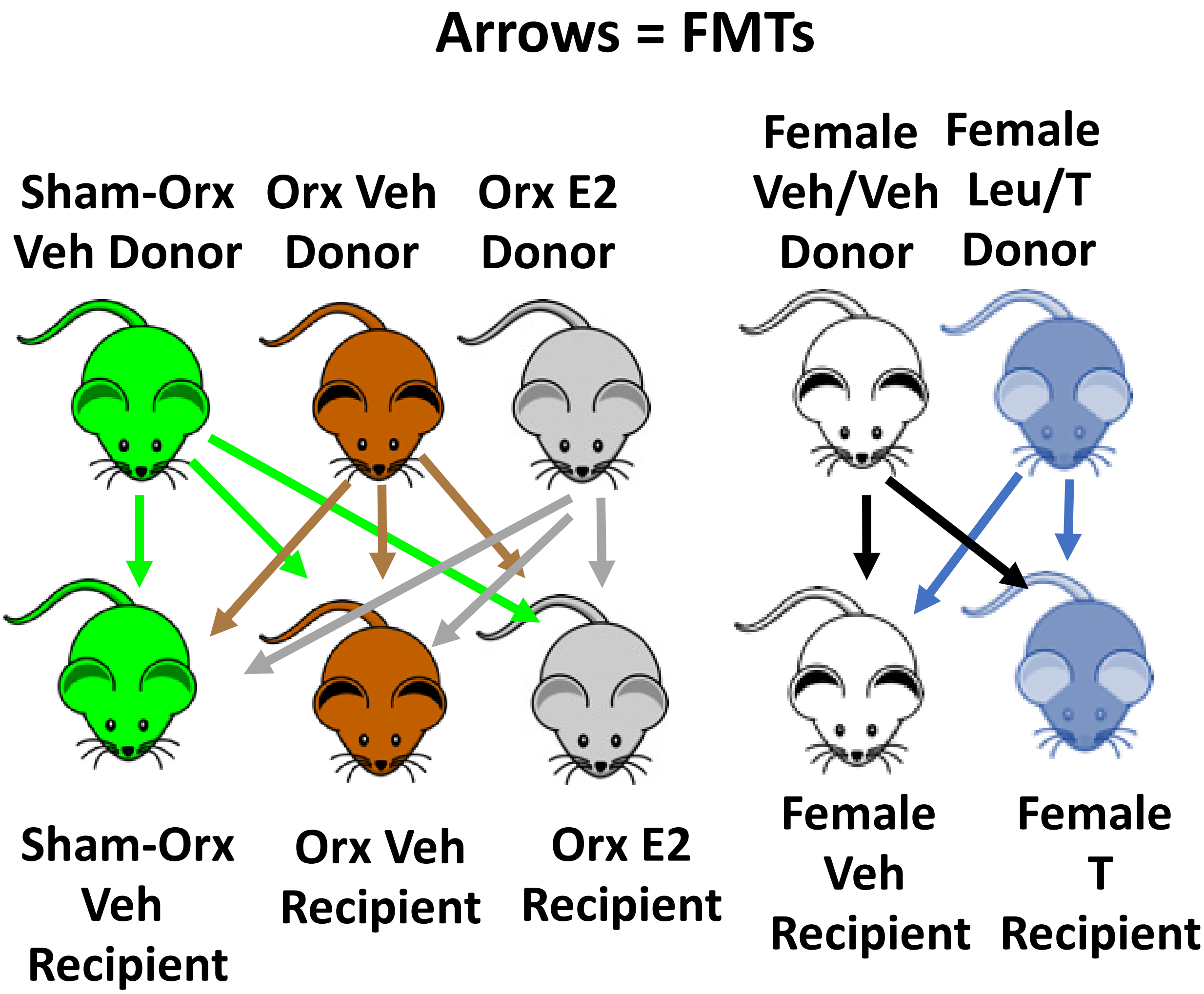

Supplemental Figure 10. Fecal microbiome transfer (FMT) experimental groups. Stool microbiome from each group of donor mice was transferred into each group of sex-matched recipient mice.

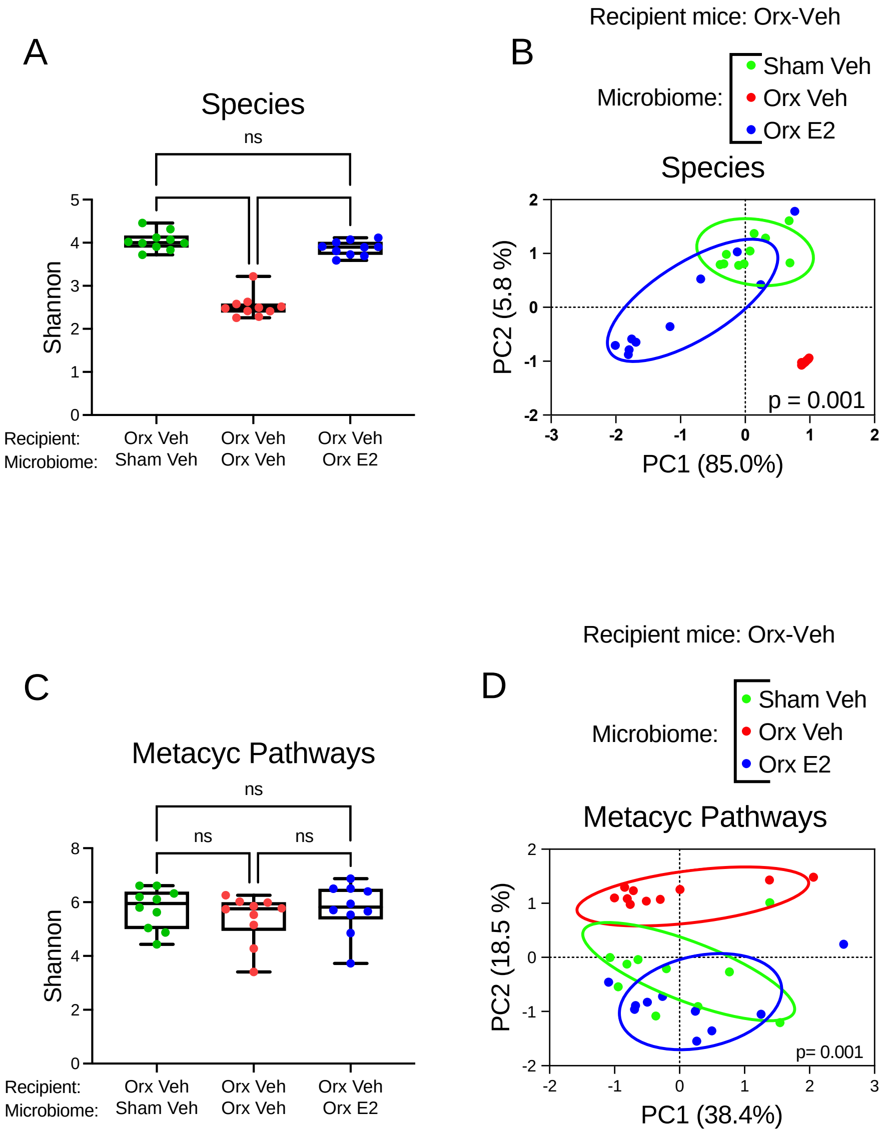

Supplemental Figure 11. Analysis of stool microbiome in male orchiectomized recipient mice. (A) Bacterial species alpha diversity analysis in GAHT treated recipient orx mice. (B) Principal coordination analysis (PCoA) of bacterial species in in GAHT treated recipient orx mice. (C) MetaCyc pathways alpha diversity analysis in GAHT treated recipient orx mice. (D) MetaCyc pathways PCoA in in GAHT treated recipient orx mice. Alpha diversity analysis was conducted by calculating the Shannon diversity index. The p-values were generated from the Wilcoxon-rank-sum test. The box shows the median and Q1-Q3 interquartile range, the bars show the minimum and maximum values. PCoA plots were based on the Bray-Curtis distance metrics. The p-values were generated from the PERMANOVA test. n = 9-10 mice per group. ** = p<0.01,**** = p<0.0001. ns= not significant.

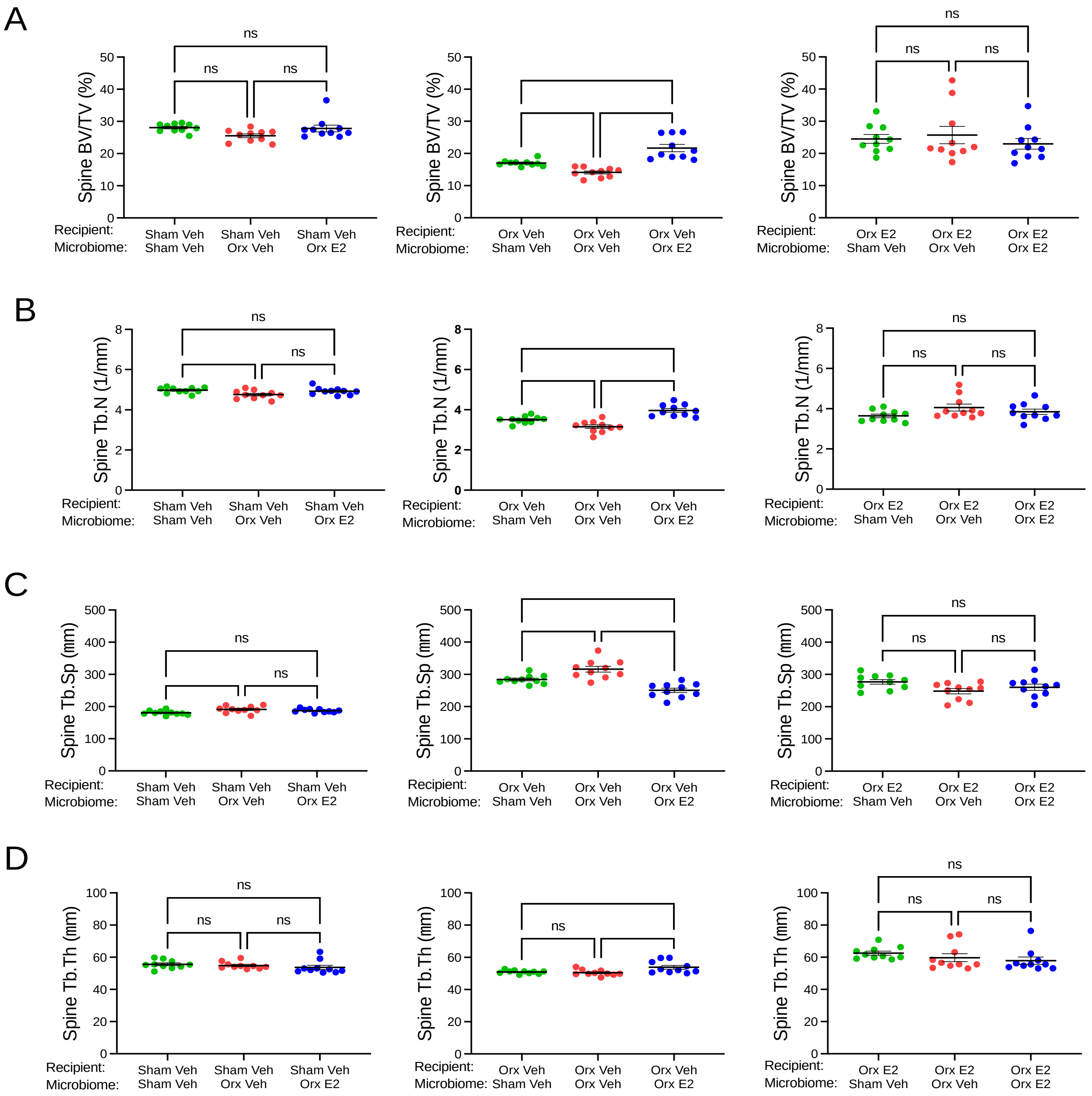

Supplemental figure 12. Microbiome transfer by fecal material transfer (FMT) altered spinal (L4) indices of trabecular volume and structure in male recipient mice. (A) BV/TV. **(B)** Tb.N. **(C)** Tb.Sp. **(D)** Tb.Th were measured by µCT at endpoint**.** Liquid suspensions of stools collected at 4 wks of GAHT from donor mice were gavaged into recipient mice. GAHT treated recipient mice were sacrificed 10 wks post treatment. n = 8-10 mice/group. All data were normally distributed and were analyzed by one-way ANOVA and post hoc tests applying Bonferroni's correction for multiple comparisons. Data were expressed as Mean ± SEM. * = p<0.05, ** = p<0.01, *** = p < 0.001 and **** = p<0.0001. ns = not significant.

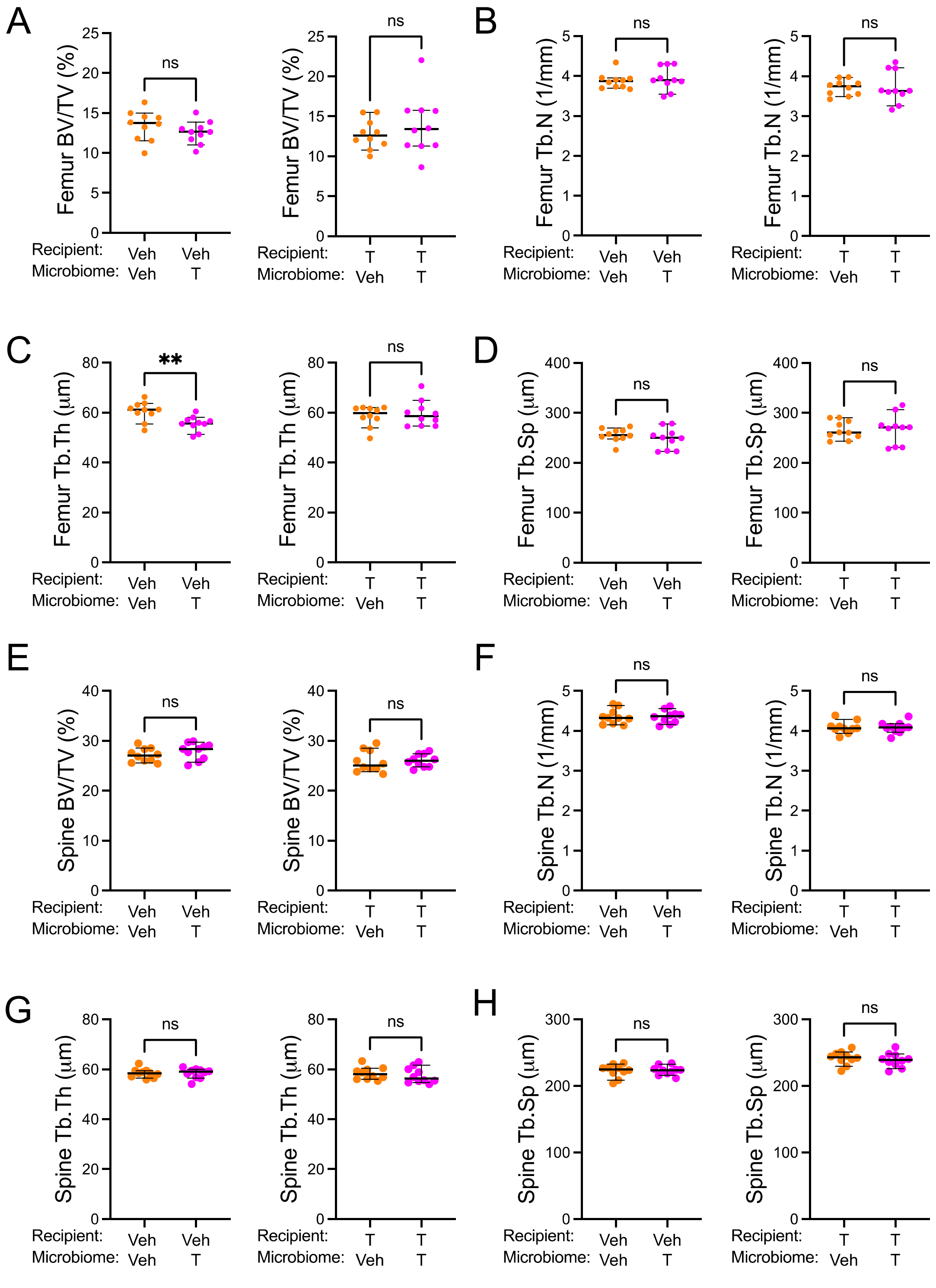

Supplemental Figure 13. Microbiome transfer by fecal material transfer (FMT) did not affect femoral and spinal (L4) indices of trabecular volume and structure in female mice. Liquid suspensions of stools collected at 4 weeks of GAHT from donor mice were gavage into recipient mice. GAHT treated recipient mice were sacrificed 10 weeks later. **(A)** Femur BV/TV. **(B)** Femur Tb.N. **(C)** Femur Tb.Sp. **(D)** Femur Tb.Th. **(E)** Spine BV/TV, **(F)** Spine Tb.N. **(G)** Spine Tb.Th. **(H)** Spine Tb.Sp. n = 10 mice/group. Mean ± SEM. All data were normally distributed and were analyzed by unpaired t-tests. ns = not significant.

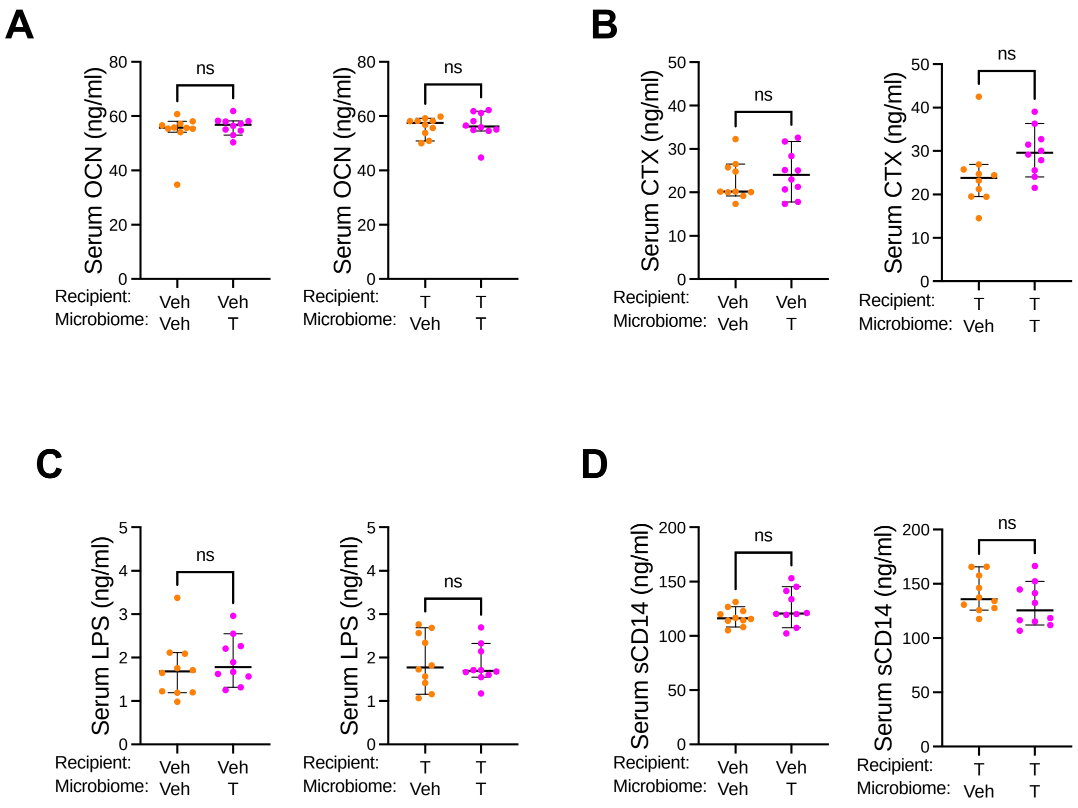

Supplemental Figure 14. Microbiome transfer by fecal material transfer (FMT) did not alter bone turnover and gut permeability in female mice. Liquid suspensions of stools collected at 4 weeks of GAHT from donor mice were gavage into recipient mice. GAHT treated recipient mice were sacrificed 10 weeks later. Serum was collected and analyzed. **(A)** Serum osteocalcin (Ocn). **(B)** Serum CTX. **(C)** Serum LPS. **(D)** Serum sCD14. n = 10 mice/group. Mean ± SEM. All data were normally distributed and were analyzed by unpaired t-tests. ns = not significant.

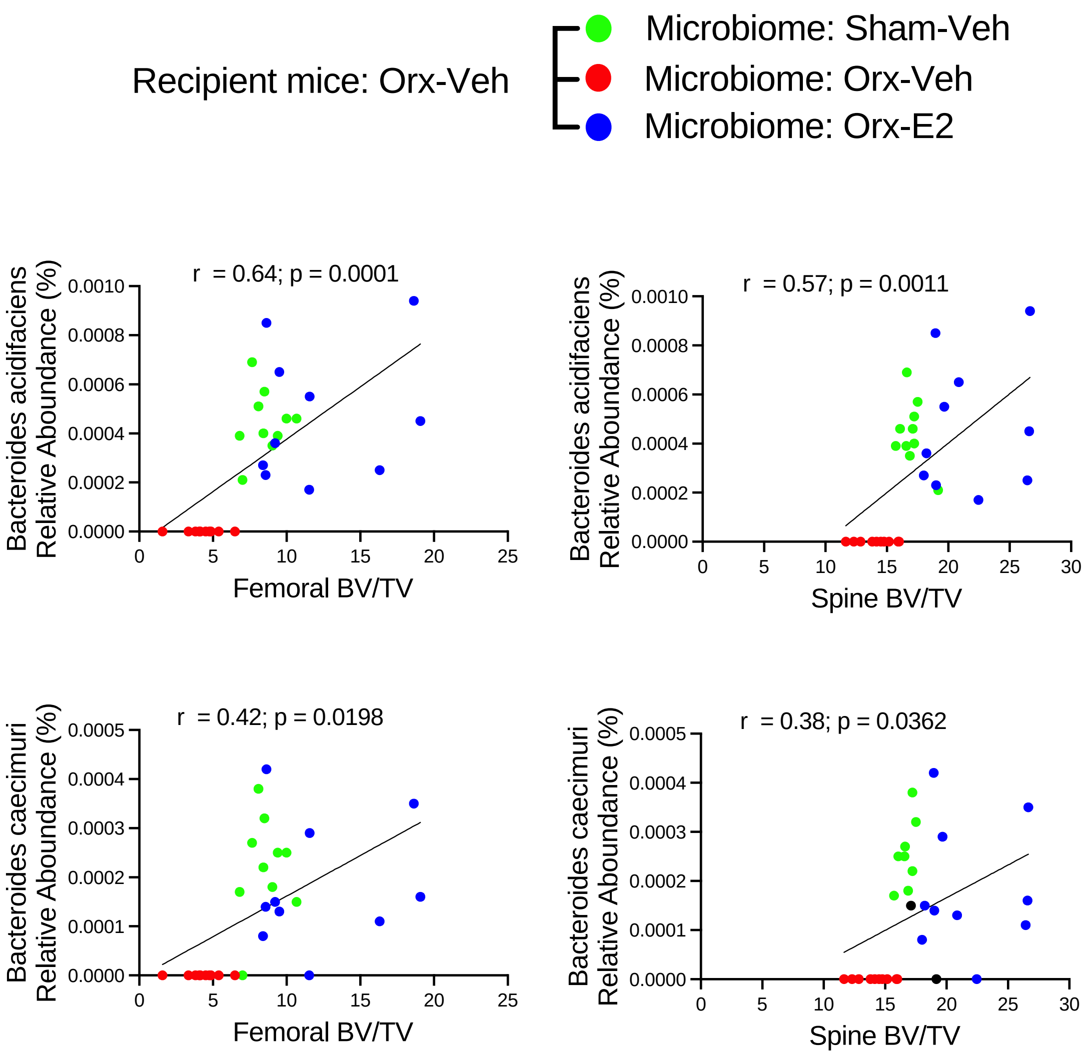

Supplemental Figure 15. Liner regressions of femoral and spinal BV/TV and the relative abundance *Bacteroides acidifaciens* and *Bacteroides caecimuri* in the stools of orx-veh recipient male. Liquid suspensions of stools collected at 4 weeks of GAHT from donor mice were gavage into recipient mice. GAHT treated recipient mice were sacrificed 10 weeks later. Stools were collected and microbiome sequenced. n = 10 mice/group.

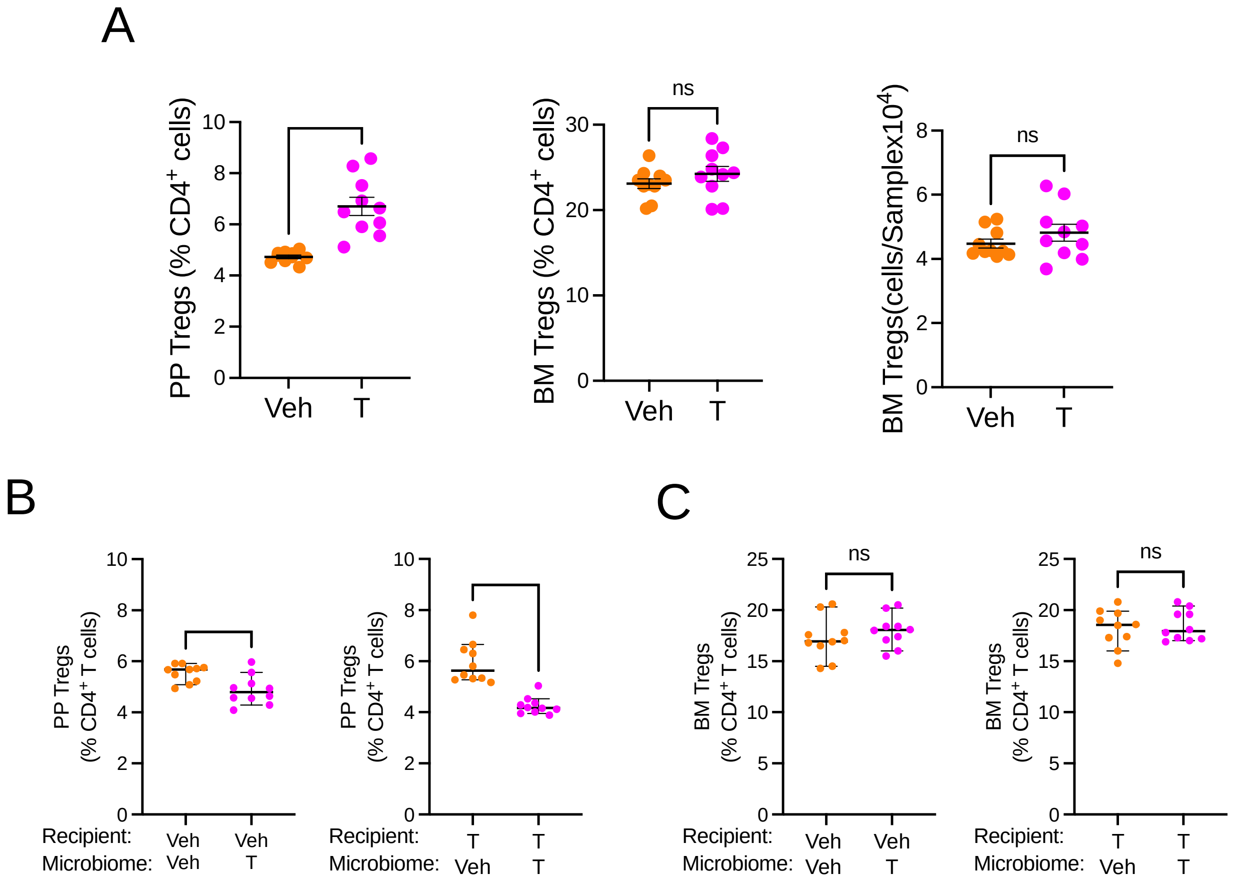

Supplemental Figure 16. Effects of GAHT and FMTs on PP and BM Tregs in female mice. (A) Relative frequency and absolute number of PP and BM Treg ($CD3^{+}CD4^{+}FOXP3^{+}$) cells in GAHT treated donor female mice. **(B)** Relative frequency of PP Tregs in female recipient mice. **(C)** Relative frequency of BM Tregs in female recipient mice. n = 10 mice/group. Mean $\pm$ SEM. All data were normally distributed and were analyzed by unpaired t-tests. ** = p<0.01 and **** = p<0.0001. ns = not significant.
